# A *Streptococcus iniae*-associated outbreak in hybrid sturgeon (*Acipenser baerii* ♀ × *Acipenser schrenckii* ♂): pathological features and transcriptomic responses to experimental challenge

**DOI:** 10.3389/fimmu.2026.1893463

**Published:** 2026-09-09

**Authors:** Luyun Ni, Hangyu Lin, Shuzhen Wang, Pengcheng Li, Yeyu Chen, Yan Liu, Jiansheng Lai, Ya Liu

**Affiliations:** 1 Fisheries Research Institute, Sichuan Academy of Agricultural Sciences (Sichuan Fisheries Research Institute), Chengdu, China; 2 College of Fisheries, Southwest University, Chongqing, China

**Keywords:** experimental challenge, full-length transcriptome, histopathology, hybrid sturgeon, immune response, *Streptococcus iniae*

## Abstract

**Introduction:**

*Streptococcus iniae* is an important bacterial pathogen of cultured fish, but its pathological effects and the host responses it induces in hybrid sturgeon remain poorly understood. This study investigated an acute disease outbreak in 4-month-old hybrid sturgeon (*Acipenser baerii* ♀ × *Acipenser schrenckii* ♂) during a high-temperature period and examined the transcriptional responses of the liver and spleen using a separate controlled challenge experiment.

**Methods:**

A dominant β-hemolytic bacterial isolate was recovered from multiple internal organs of naturally diseased fish and identified using phenotypic characterization, 16S rRNA gene sequencing and phylogenetic analysis, and a species-specific PCR assay targeting the *lctO* gene. Histopathology and transmission electron microscopy were used to characterize tissue and subcellular alterations. In the controlled challenge, liver and spleen samples were collected at 48 h post-injection and analyzed using PacBio single-molecule real-time and Illumina RNA sequencing.

**Results:**

Affected fish exhibited abnormal swimming, a swollen and hyperemic anus, and pronounced gas and fluid accumulation in the spiral valve intestine and swim bladder. The isolate was identified as *S. iniae*. Histopathological examination revealed hepatic congestion and necrosis, splenic white pulp atrophy, intestinal mucosal exfoliation, myocarditis, and gill injury. Transmission electron microscopy showed coccoid bacterial structures in several tissues, together with mitochondrial swelling, cristae disruption, microvillar damage, and autophagosome-like structures. PacBio sequencing generated 120,215 non-redundant transcripts, with 94.4% completeness according to BUSCO analysis, providing a full-length transcript reference for short-read expression analysis. Gene set enrichment analysis showed negative enrichment of the fat digestion and absorption pathway and positive enrichment of the apoptosis pathway in the liver. In the spleen, the Toll and Imd and IL-17 signaling pathways were positively enriched. RT-qPCR analysis of eight selected transcripts showed expression directions consistent with the RNA-seq results.

**Discussion:**

These findings characterize the pathological features of *S. iniae*-associated disease in hybrid sturgeon and provide a transcript-level view of the liver and spleen responses following controlled experimental challenge.

## 1 Introduction

Sturgeons, representing one of the most primitive lineages of actinopterygian fishes, hold profound ecological, evolutionary, and commercial value worldwide (1). Driven by the escalating global demand for flesh and high-value caviar, sturgeon aquaculture has undergone significant expansion over the past few decades, particularly in China (2). However, the rapid intensification of farming practices, combined with environmental stressors exacerbated by global climate warming, has rendered these ancient species increasingly vulnerable to infectious diseases (3). Among these, bacterial pathogens pose a continuous threat to the sustainable development of the sturgeon industry, often resulting in mass mortalities and severe economic losses (4, 5).

*Streptococcus iniae* is a versatile, gram-positive, beta-hemolytic pathogen that poses a globally prevalent threat to both marine and freshwater aquaculture (6). It infects a wide range of economically important teleosts, such as Nile tilapia (*Oreochromis niloticus*) (7), golden pompano (*Trachinotus ovatus*) (8), hybrid striped bass (*Morone chrysops* × *Morone saxatilis*) (9), and rainbow trout (*Oncorhynchus mykiss*) (10), typically eliciting systemic hemorrhagic septicemia and pericarditis. Furthermore, *S. iniae* is recognized as a zoonotic agent capable of causing severe public health concerns, including human sepsis and meningitis (11). In recent years, outbreaks of streptococcosis associated with *S. iniae* have been increasingly documented in various sturgeon species across China, including Siberian sturgeon (*A. baerii*) and Chinese sturgeon (*Acipenser sinensis*), and hybrid sturgeon (12–14). Notably, Wang et al. previously isolated *S. iniae* from naturally diseased hybrid sturgeon of the same parental combination examined in the present study and confirmed its pathogenicity through experimental infection (14). Therefore, the present study does not represent the first report of *S. iniae* infection in this hybrid. However, previous studies focused primarily on pathogen isolation, pathogenicity, and antimicrobial susceptibility, whereas the multi-organ histopathological and ultrastructural features of the disease, together with tissue-specific host transcriptional responses, remain insufficiently characterized.

In June 2025, an acute disease outbreak with a daily mortality rate of approximately 10% occurred in farmed 4-month-old hybrid sturgeons (*A. baerii* ♀ × *A. schrenckii* ♂) during a high-temperature period, with an average water temperature of 25-27 °C in Sichuan Province, China. The affected fish exhibited abnormal swimming behavior, systemic hemorrhagic lesions, and pronounced gas and fluid accumulation in the spiral valve intestine. To characterize this outbreak, a dominant bacterial isolate was recovered from diseased fish and identified as *S. iniae* through bacteriological, phenotypic, and molecular analyses. Histopathology and transmission electron microscopy were subsequently used to characterize tissue and subcellular alterations in the naturally diseased fish. In a separate controlled challenge experiment, the transcriptomic responses of the liver and spleen were investigated at 48 h post-injection using an integrated PacBio single-molecule real-time (SMRT) and Illumina sequencing strategy. This study aimed to characterize the pathological features associated with the outbreak and to identify candidate host transcriptional responses to experimental *S. iniae* challenge.

## 2 Materials and methods

### 2.1 Clinical observation

Twelve symptomatic hybrid sturgeons (*A. baerii* ♀ × *A. schrenckii* ♂) were collected from an intensive sturgeon farm in Sichuan Province, China, with a mean body weight of 27.74 ± 3.43 g. Apparently healthy fish from the outbreak farm were not selected because early-stage or subclinical infection could not be excluded. Instead, nine age- and size-matched clinically healthy hybrid sturgeon were used as healthy morphological references for gross, histopathological, and ultrastructural comparisons. According to the farming records, the diseased and healthy fish originated from the same production batch and were maintained using the same commercial diet and water source. The healthy fish exhibited normal swimming and feeding behavior, with no apparent external lesions or parasitic infestation. To comply with animal welfare standards and ensure high-quality sampling, all sturgeons were first deeply anesthetized and then humanely euthanized by prolonged immersion in MS-222 solution at a concentration of 200 mg/L prior to systematic autopsy. Death was confirmed by the absence of opercular movement and response to external stimuli before dissection. The clinical characteristics and macroscopic pathological changes in the internal organs were then meticulously investigated and recorded.

### 2.2 Bacterial isolation and identification

Before dissection, the body surface of each diseased fish was disinfected with 75% ethanol. Liver, spleen, and heart tissues were aseptically streaked onto blood agar plates (HuanKai Microbial, Guangdong, China) and incubated at 28 °C for 48 h. The medium composition is provided in **Supplementary Table 1**. The dominant colony morphotype consistently recovered from multiple organs was purified by repeated subculture and selected for identification. Bacterial genomic DNA was extracted using a Universal Genomic DNA Kit (CWBIO, Beijing, China). The *16S rRNA* gene was amplified using universal primers 27F (5′-AGAGTTTGATCCTGGCTCAG-3′) and 1492R (5′-TACGGCTACCTTGTTACGACTT-3′) (15). A 50 μL reaction system was employed, containing 25 μL of 2×Taq Plus MasterMix (CWBIO), 0.2 μg of template DNA, and 2 μL of each 10 μM primer. The thermal cycling profile included an initial denaturation (94 °C, 2 min), followed by 35 cycles of 94 °C (30 s), 55 °C (30 s), and 72 °C (45 s), with a final extension at 72 °C for 2 min. Species-specific PCR targeting the *S. iniae lctO* gene was performed using primers *lctO*-F (5′-AAGGGGAAATCGCAAGTGCC-3′) and *lctO*-R (5′-ATATCTGATTGGGCCGTCTAA-3′) as described by Mata et al. (16). The 16S rRNA and *lctO* amplicons were sequenced by Sangon Biotech (Chengdu, China) and analyzed using BLASTn against the NCBI nucleotide database. For phylogenetic analysis, closely related 16S rRNA gene sequences were aligned and a maximum-likelihood tree was constructed in MEGA 11 using the Tamura three-parameter model with gamma-distributed rate variation and 1,000 bootstrap replicates. Positions with less than 75% site coverage were removed by partial deletion. *Streptococcus dysgalactiae* and *S. lutetiensis* were included for comparison. Identification of the isolate was based on 16S rRNA sequence and phylogenetic analyses together with the species-specific *lctO* assay.

### 2.3 Morphological characterization of the bacteria

The morphological features of the isolated bacteria were characterized using both Scanning Electron Microscopy (SEM) and TEM. For surface morphology analysis, the bacterial cells were harvested from the exponential growth phase by centrifugation at 5000 rpm for 10 min. The pellets were washed three times with 0.1 M phosphate-buffered saline (PBS, pH 7.2) and fixed with 2.5% (v/v) glutaraldehyde at 4 °C for 12 h. After fixation, the samples were dehydrated through a graded ethanol series (30%, 50%, 70%, 80%, 90%, and 100%) for 15 min at each step. The dehydrated specimens were then subjected to critical point drying and sputter-coated with gold-palladium. Observations were conducted using a JSM-IT700HR SEM (JEOL Ltd., Tokyo, Japan). To observe the internal ultrastructure, the bacterial samples were fixed in 2.5% glutaraldehyde as described above and post-fixed with 1% osmium tetroxide for 2 h. Following dehydration in a graded series of ethanol and acetone, the samples were embedded in epoxy resin. Ultra-thin sections (60–80 nm) were cut using an ultramicrotome with a diamond knife and subsequently stained with uranyl acetate and lead citrate. The prepared sections were examined under JEM-1400-FLASH TEM (JEOL Ltd., Tokyo, Japan).

### 2.4 Physiological and biochemical characterization and antimicrobial susceptibility testing

The phenotypic characteristics of the purified isolate were analyzed using bacterial biochemical identification kits (Hangzhou Microbial Reagent Co., Ltd., Hangzhou, China). Pure cultures were inoculated into various biochemical reaction tubes to evaluate growth temperature ranges (10 °C and 45 °C), salt tolerance (6.5% and 8.0% NaCl), and carbon source fermentation (including *D*-mannitol, sorbitol, trehalose, arabinose, raffinose, ribose, and lactose). Key enzyme activities, such as oxidase and catalase, along with specific metabolic pathways—including arginine hydrolysis, esculin utilization (esculin hydrolysis), the Voges-Proskauer (V-P) test, and hemolysis type—were also determined. The inoculated tubes were incubated at 28 °C for 24–48 h, and physiological and biochemical characteristics were judged based on color changes in each reaction tube.

The antibiotic susceptibility of the isolate was evaluated using the Kirby-Bauer (K-B) disk diffusion method with antibiotic disks (Hangzhou Microbial Reagent Co., Ltd., Hangzhou, China). A total of 20 antibiotics spanning 10 categories were tested, including penicillins (ampicillin), cephalosporins (cefoperazone and ceftriaxone), tetracyclines (minocycline and tetracycline), aminoglycosides (streptomycin, kanamycin, gentamicin, tobramycin, spectinomycin, and amikacin), quinolones (enrofloxacin and levofloxacin), nitrofurans (nitrofurantoin), macrolides (erythromycin, clarithromycin, and midecamycin), polypeptides (polymyxin B), lincosamides (clindamycin), and chloramphenicols (florfenicol). Pure bacterial cultures were prepared as suspensions with a concentration of approximately 1.5×10^8^ CFU/mL (0.5 McFarland standard) and uniformly spread onto Muller Hinton agar plates (Hopebio, Qingdao, China). The assay was performed in triplicate. The diameters of the inhibition zones were measured and reported in millimeters. Because species-specific clinical breakpoints are unavailable for fish-derived *S. iniae* for most of the tested antimicrobials, inhibition-zone diameters were reported as the primary results. Susceptible, intermediate, and resistant categories were provisionally assigned according to the interpretive criteria supplied by the disk manufacturer, with reference to relevant CLSI guidelines for bacteria isolated from aquatic animals where applicable (17).

### 2.5 Histopathological and ultrastructural analysis

Three naturally diseased hybrid sturgeon and three clinically healthy reference fish were used for histopathological and ultrastructural examinations. Gill, heart, liver, spleen, and spiral valve intestinal tissues were collected from naturally diseased hybrid sturgeon and clinically healthy reference fish. For histopathological examination, the tissue samples were immediately fixed in 4% paraformaldehyde for 24 h, followed by dehydration through a graded ethanol series, clearing in xylene, and embedding in paraffin wax. One 5-μm-thick section was prepared from each tissue of each fish using a rotary microtome and stained with hematoxylin and eosin (H&E). Each section was digitized by whole-slide scanning (DM1000; Leica, Germany), and the entire tissue section was examined rather than a predetermined number of microscopic fields. Histological alterations in the diseased fish were descriptively compared with the corresponding tissue structures in the healthy reference fish. For ultrastructural observation using TEM, small tissue blocks (approximately 1 mm^3^) were rapidly immersed in 2.5% glutaraldehyde and fixed at 4 °C. These samples were then rinsed with 0.1 M PBS, post-fixed in 1% osmium tetroxide, dehydrated in a graded acetone series, and embedded in epoxy resin. One ultrathin section, 60–80 nm in thickness, was prepared from each tissue of each fish using an ultramicrotome and stained with uranyl acetate and lead citrate. The sections were examined by transmission electron microscopy to document alterations. The histopathological and ultrastructural examinations were descriptive. The group identities were known during evaluation, and no blinded semiquantitative lesion scoring or inferential statistical comparison was performed.

### 2.6 Artificial challenge experiment

A controlled challenge experiment was conducted to obtain tissues for transcriptome sequencing. Based on a preliminary dose-ranging experiment, a bacterial suspension concentration of 1.5 × 10^7^ CFU/mL was selected for the controlled challenge because it corresponded approximately to the median lethal dose under the present experimental conditions. Healthy hybrid sturgeons were randomly assigned to an *S. iniae*-challenged group and a PBS-injected control group, with 15 fish per group distributed equally among three independent tanks. Fish were intraperitoneally injected with 200 μL of either the bacterial suspension (3.0 × 10^6^ CFU/fish) or sterile PBS and maintained at 20 °C. Clinical signs and mortality were monitored throughout the experiment.

At 48 h post-injection, one fish was randomly selected from each tank, resulting in three biological replicates per treatment group. The fish were euthanized as described above, and bacterial re-isolation from the internal organs of the challenged fish was confirmed using colony morphology and the *S. iniae*-specific *lctO* PCR assay. Liver and spleen tissues were collected and stored at −80 °C after snap-freezing in liquid nitrogen. Each tissue sample was processed independently for Illumina RNA-seq, yielding 12 libraries. Aliquots of all liver and spleen RNA samples were pooled into one composite sample for PacBio SMRT sequencing to generate the full-length transcript reference; this pooled library was not treated as a biological replicate in differential expression analysis.

### 2.7 PacBio SMRT and Illumina transcriptome sequencing

Total RNA was isolated from the spleen and liver tissues of challenged and PBS-control hybrid sturgeons utilizing the TRIzol reagent (Invitrogen, Carlsbad, CA, USA) in accordance with the provided instructions. RNA degradation and potential DNA contamination were evaluated by agarose gel electrophoresis. RNA purity, concentration, and integrity were further assessed using a NanoDrop 2000 spectrophotometer (Thermo Fisher Scientific, Wilmington, DE, USA), a Qubit 2.0 fluorometer (Thermo Fisher Scientific), and an Agilent 2100 Bioanalyzer (Agilent Technologies, Palo Alto, CA, USA), respectively. Only RNA samples that met the quality requirements were used for library construction.

For full-length transcript sequencing, a single composite sample representing the collected tissues from the challenged and PBS-control fish was prepared for PacBio library construction. Full-length cDNA was synthesized and amplified using the PacBio Kinnex full-length RNA workflow, which employs MAS-Seq concatenation to increase sequencing throughput. After purification, the cDNA products were used for Kinnex array formation, nuclease treatment, and SMRTbell library preparation according to the manufacturer’s instructions. The resulting library was sequenced on the PacBio Revio platform (Pacific Biosciences, Menlo Park, CA, USA). The PacBio dataset was used to generate a nonredundant full-length transcript reference and was not treated as a biological replicate in differential expression analysis.

For Illumina RNA-seq, the qualified RNA samples were processed separately without pooling. Polyadenylated mRNA was enriched using oligo(dT) magnetic beads and fragmented into short fragments. First-strand cDNA was synthesized using random primers, followed by second-strand cDNA synthesis. The resulting double-stranded cDNA was purified, end-repaired, A-tailed, and ligated to Illumina sequencing adapters. After size selection and PCR amplification, the libraries were sequenced on an Illumina HiSeq 4000 platform by Gene Denovo Biotechnology Co., Ltd. (Guangzhou, China) to generate 150-bp paired-end reads. For the present study, downstream transcriptomic analyses were restricted to the 12 independently prepared liver and spleen libraries, comprising three biological replicates for each tissue and treatment combination.

### 2.8 Transcriptome data processing and bioinformatic analyses

PacBio sequencing data were processed using SMRT Link v13.1. Circular consensus sequences with a quality value greater than 20 were retained as HiFi reads (18). Primer and barcode sequences were removed using Lima in Iso-Seq v4.3.0, after which poly(A) tails and concatemeric sequences were identified and removed using the refine module to generate full-length non-concatemer reads. Highly similar full-length reads were clustered using the cluster2 module to generate high-quality consensus transcripts. Redundant sequences were subsequently collapsed using CD-HIT v4.8.1 (19), and the resulting nonredundant transcript set was used as the reference transcriptome. The completeness of the reference transcriptome was evaluated using BUSCO v5.8.2 (20). Functional annotation was performed by comparing the nonredundant transcript sequences against the NCBI nonredundant protein, Swiss-Prot, KEGG, and KOG databases using DIAMOND with an E-value threshold of 1×10^−5^ (21). Gene Ontology annotations were assigned based on the corresponding homologous protein annotations.

Raw Illumina reads were filtered using fastp v0.18.0 (22). Reads containing adapter sequences, reads with more than 10% unknown nucleotides, and reads in which more than 50% of the bases had a quality score of ≤20 were removed. The clean paired-end reads from each library were mapped to the PacBio-derived nonredundant reference transcriptome, and transcript abundance was estimated using RSEM (23). RSEM-derived raw read counts were used for differential expression analysis, whereas transcripts per million values were used to summarize expression abundance and for data visualization. Sample correlations and principal component analysis were used to evaluate consistency among biological replicates and the overall relationships among samples.

Differential expression analyses were performed separately for the liver and spleen using DESeq2 (24). Within each tissue, the three challenged fish, each originating from an independent tank, were compared with the three corresponding PBS-injected control fish. The DESeq2 models were fitted using the raw count matrix. *P*-values were adjusted for multiple testing using the Benjamini-Hochberg procedure. Transcripts with a false discovery rate of <0.05 and an absolute log2 fold change of >1 were considered differentially expressed. Gene Ontology and KEGG pathway enrichment analyses were performed for the differentially expressed transcripts (DETs) using a hypergeometric test, followed by false discovery rate correction. Terms or pathways with an adjusted *P*-value of <0.05 were considered significantly enriched. Gene set enrichment analysis (GSEA) was additionally conducted using the complete expression dataset rather than a preselected list of differentially expressed transcripts (25). Transcripts were ranked according to the Signal-to-Noise metric, and enrichment scores, normalized enrichment scores, nominal P-values, and false discovery rate q-values were calculated using gene-set permutations. Gene sets with an absolute normalized enrichment score of >1, a nominal *P*-value of <0.05, and a false discovery rate q-value of <0.25 were considered significantly enriched. All differential expression and enrichment analyses were conducted separately for the liver and spleen.

### 2.9 Quantitative real-time PCR validation

Eight transcripts, including ferritin heavy chain 1 (*FTH1*), glutathione peroxidase 1 (*GPX1*), peroxiredoxin 5 (*PRDX5*), DNA damage-regulated autophagy modulator 1 (*DRAM1*), matrix metallopeptidase 13 (*MMP13*), CD74 molecule (*CD74*), complement C1q C chain (*C1QC*), and fatty acid-binding protein 7 (*FABP7*), were selected to evaluate the consistency of the RNA-seq results using quantitative real-time PCR (RT-qPCR). These transcripts were selected to represent different expression directions and fold-change magnitudes, as well as major functional categories identified by RNA-seq, while also allowing the design of gene-specific primers. Gene-specific primers for RT-qPCR validation were listed in **Supplementary Table 2**. Total RNA from three challenged and three PBS-control fish per tissue was reverse-transcribed into cDNA. RT-qPCR was performed using an ABI 7500 Fast Real-Time PCR System (Applied Biosystems, Foster City, CA, USA) with 2× SG Fast qPCR Master Mix (Sangon Biotech, Shanghai, China). The thermal cycling conditions were initiated with a denaturation step at 95 °C for 3 min, followed by 45 cycles of 95 °C for 15 s, 60 °C for 30 s, and 72 °C for 30 s. In accordance with the Minimum Information for Publication of Quantitative Real-Time PCR Experiments (MIQE) guidelines (26), the expression stability of two potential reference genes *β-actin* and *EF1α*, was evaluated using the NormFinder program. After identifying the most stable internal control, the relative mRNA quantification of the target genes was determined using the 2^-ΔΔCt^ method (27). For comparison with RNA-seq, the RT-qPCR results were expressed as log2 fold changes, calculated as −ΔΔCt. Values are presented as the mean ± standard deviation of three biological replicates. RNA-seq and RT-qPCR fold changes were compared according to their direction and relative magnitude. A bar plot for RT-qPCR validation was generated using GraphPad Prism 9.

## 3 Results

### 3.1 Clinical and gross pathological features

**Figure 1:**
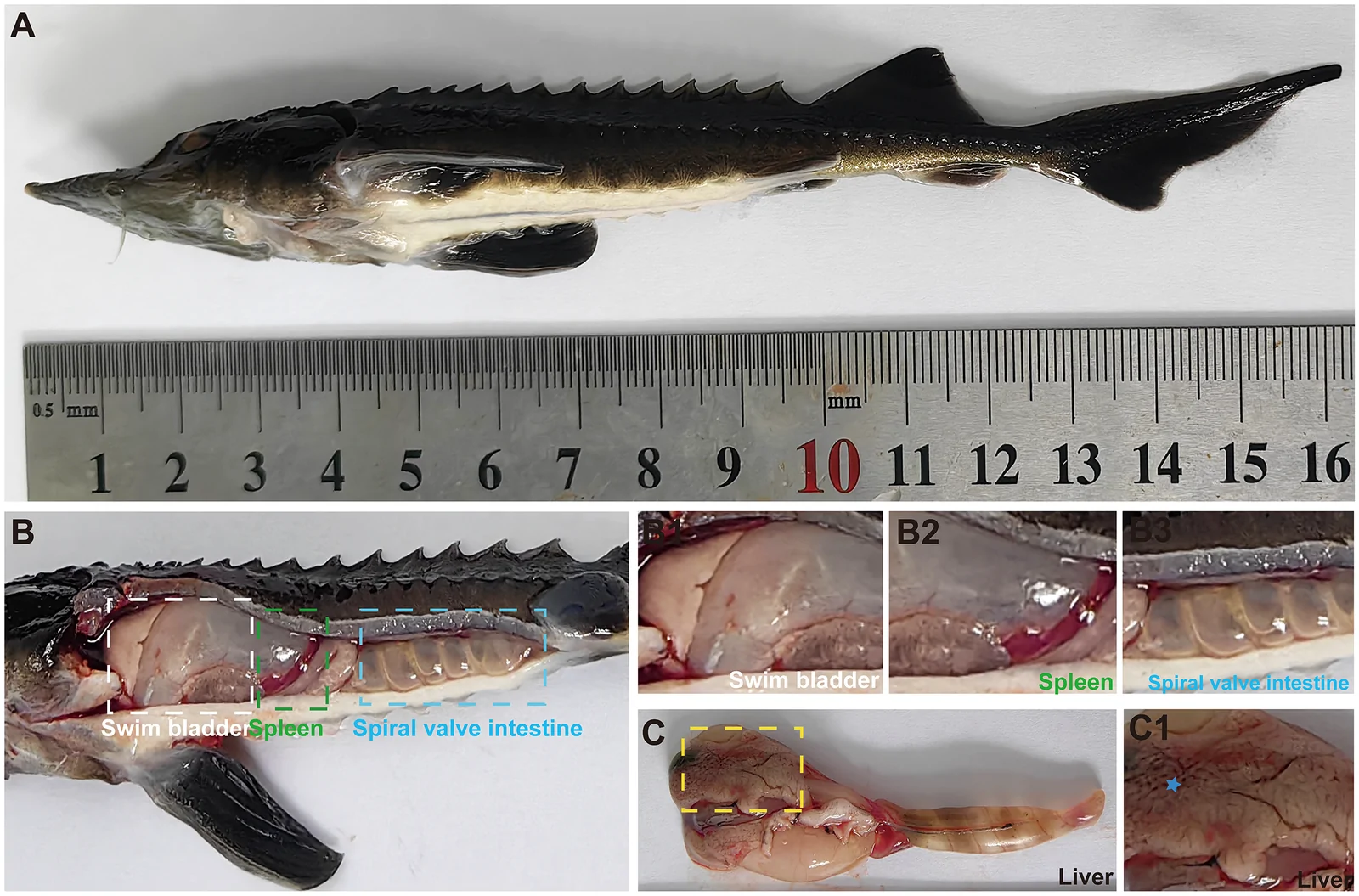
Clinical features and gross pathological lesions of diseased hybrid sturgeons. **(A)** External morphology of the diseased sturgeon. **(B)** Anatomical features of abdominal viscera. **(B1–B3)** Distended and transparent swim bladder (white box), spleen (green box), and spiral valve intestine (blue box) containing turbid bubbles. **(C, C1)** Liver showing a “mottled” appearance (yellow box) with distinct multifocal hemorrhages (blue star).

In June 2025, an acute disease outbreak occurred in 4-month-old hybrid sturgeons (*A. baerii* ♀ × *A. schrenckii* ♂) at an intensive farm in Sichuan Province, China, characterized by a daily mortality rate of approximately 10%. The diseased fish exhibited distinct abnormal behaviors, most notably “backstroke” or upside-down swimming. Gross examination revealed marked distension of the gastrointestinal tract, particularly the spiral valve intestine. The stomach and intestinal lumen contained little or no feed but showed abundant gas bubbles and turbid fluid. The liver exhibited a mottled appearance with multifocal hemorrhagic lesions, and the spleen was enlarged. The blood of affected fish appeared dark red. In contrast, the clinically healthy reference fish remained active, fed normally, and showed no comparable external abnormalities or gross lesions in the examined internal organs (**Figure 1**).

### 3.2 Isolation and identification of pathogen

**Figure 2:**
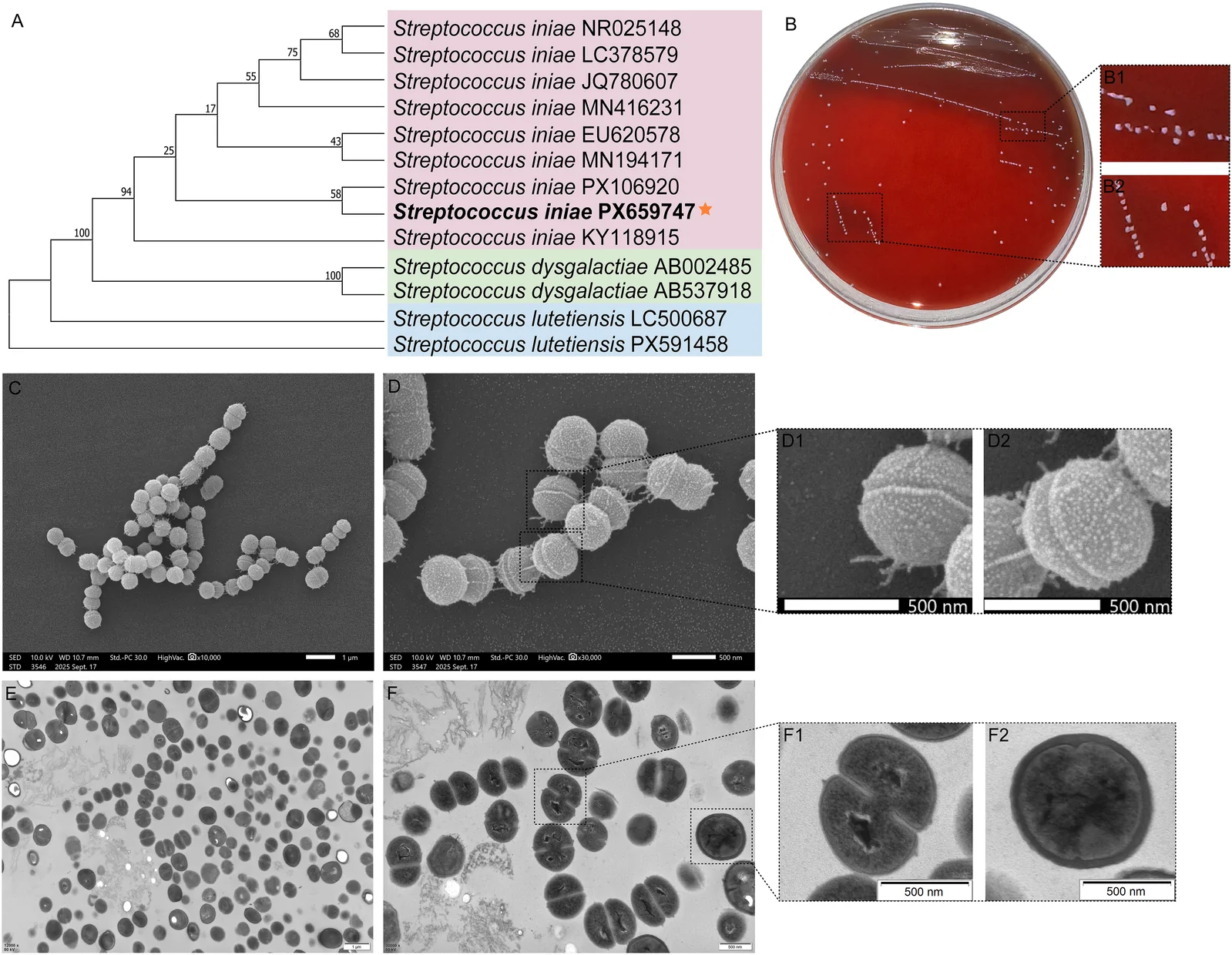
Morphology observation and phylogenetic tree of isolated S. iniae (strain PX659747). **(A)** Phylogenetic tree constructed using ML method based on *16S rRNA* sequences; **(B–B2)** Colonial morphology on blood agar; **(C, D–D2)** SEM images showing the chain-like arrangement and surface morphology of the isolate; **(E, F–F2)** TEM images revealing the cell wall ultrastructure and binary fission.

A dominant bacterial strain was successfully isolated from the liver and spleen of symptomatic hybrid sturgeons using aseptic sampling and cultivation techniques. PCR amplification using the universal primers 27F and 1492R yielded an approximately 1.5-kb product. Following bidirectional Sanger sequencing, sequence assembly, and quality trimming, a 1,095-bp partial *16S rRNA* gene sequence was obtained and deposited in GenBank under accession number PX659747. BLASTn analysis showed that the sequence shared 98.87% identity with independent *S. iniae* reference sequences, with 97% query coverage. In the phylogenetic tree, PX659747 clustered within the *S. iniae* clade, which was supported by a bootstrap value of 94%, and was separated from the *S. dysgalactiae* and *S. lutetiensis* reference sequences (**Figure 2A**). In addition, amplification and sequence analysis of the species-specific *lctO* gene provided further support for identifying the isolate as *S. iniae*. The colonial morphology was characterized by small, circular, and grayish-white colonies with well-defined margins following incubation at 28 °C on blood agar. These colonies displayed a β-hemolytic phenotype (**Figures 2B–B2**). Morphological identification was further corroborated through advanced imaging. SEM revealed that the bacterial cells were spherical or ovoid cocci, measuring approximately 0.5–0.8 μm in diameter, and arranged in short to medium-length chains (**Figures 2C, D–D2**). Furthermore, TEM displayed ultrastructural features, including a thick, electron-dense cell wall and active binary fission (**Figures 2E, F–F2**).

### 3.3 Physiological and biochemical characteristics and antimicrobial susceptibility

The physiological and biochemical characteristics of the *S. iniae* isolate are summarized in **Supplementary Table 3**. The isolate was capable of growth at 10 °C but showed no growth at 45 °C or in media containing 6.5% and 8.0% NaCl. Regarding carbon source utilization, the strain fermented *D*-mannitol and trehalose, while yielding positive results for both arginine hydrolysis and esculin utilization. In contrast, fermentation tests for sorbitol, arabinose, raffinose, ribose, and lactose were negative. Furthermore, the isolate lacked oxidase, catalase, and V-P metabolic activities, and its hemolytic activity was classified as β-type, consistent with the initial colonial observations.

The inhibition-zone diameters produced by the tested antimicrobial agents are presented in **Supplementary Table 4**. Based on the provisional interpretive criteria described in the Methods, the isolate was classified as susceptible to ampicillin, cefoperazone, ceftriaxone, minocycline, streptomycin, kanamycin, tobramycin, clarithromycin, and florfenicol. Ceftriaxone produced the largest inhibition zone, with a diameter of 37 mm. Intermediate responses were recorded for tetracycline, gentamicin, nitrofurantoin, erythromycin, midecamycin, and polymyxin B, whereas the isolate was classified as resistant to enrofloxacin, levofloxacin, spectinomycin, and amikacin. These results describe the *in vitro* antimicrobial susceptibility profile of the isolate and should not be interpreted directly as evidence of therapeutic efficacy in infected hybrid sturgeon.

### 3.4 Histopathological changes in naturally diseased hybrid sturgeon

**Figure 3:**
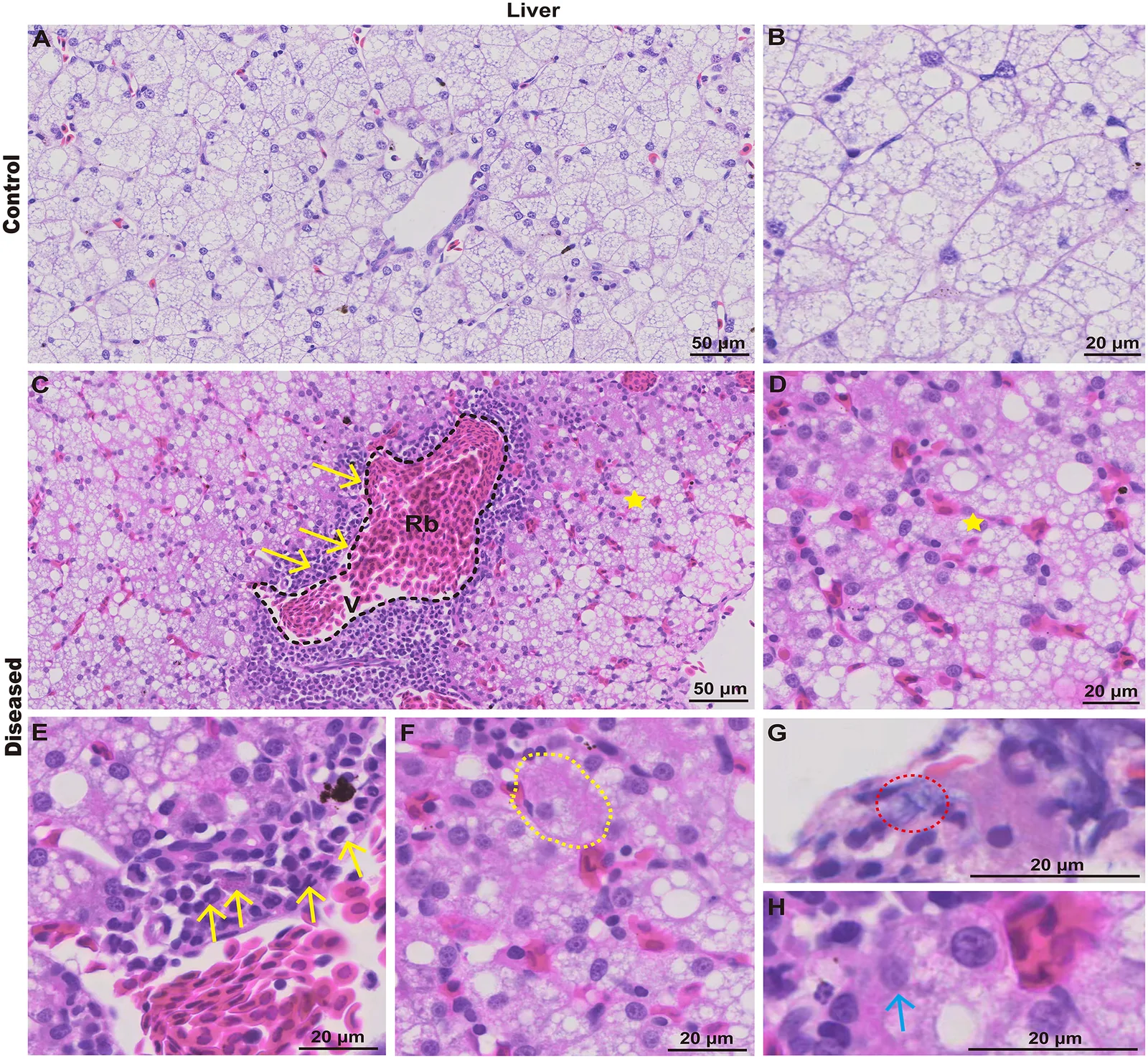
Histopathological examination of the liver in hybrid sturgeons. **(A, B)** Control group showing regular hepatic cord structures and normal cell morphology. **(C, D)** Diseased hybrid sturgeon exhibiting vascular (V) dilation, massive red blood cell (Rb) congestion (Black dashed box and yellow star). **(E)** Prominent perivascular inflammatory infiltration. **(F–H)** Diseased group showing vacuolar degeneration, focal necrosis (yellow dashed circle), and bacterial clusters (red dashed circle); nuclear pyknosis is indicated by the blue arrow.

**Figure 4:**
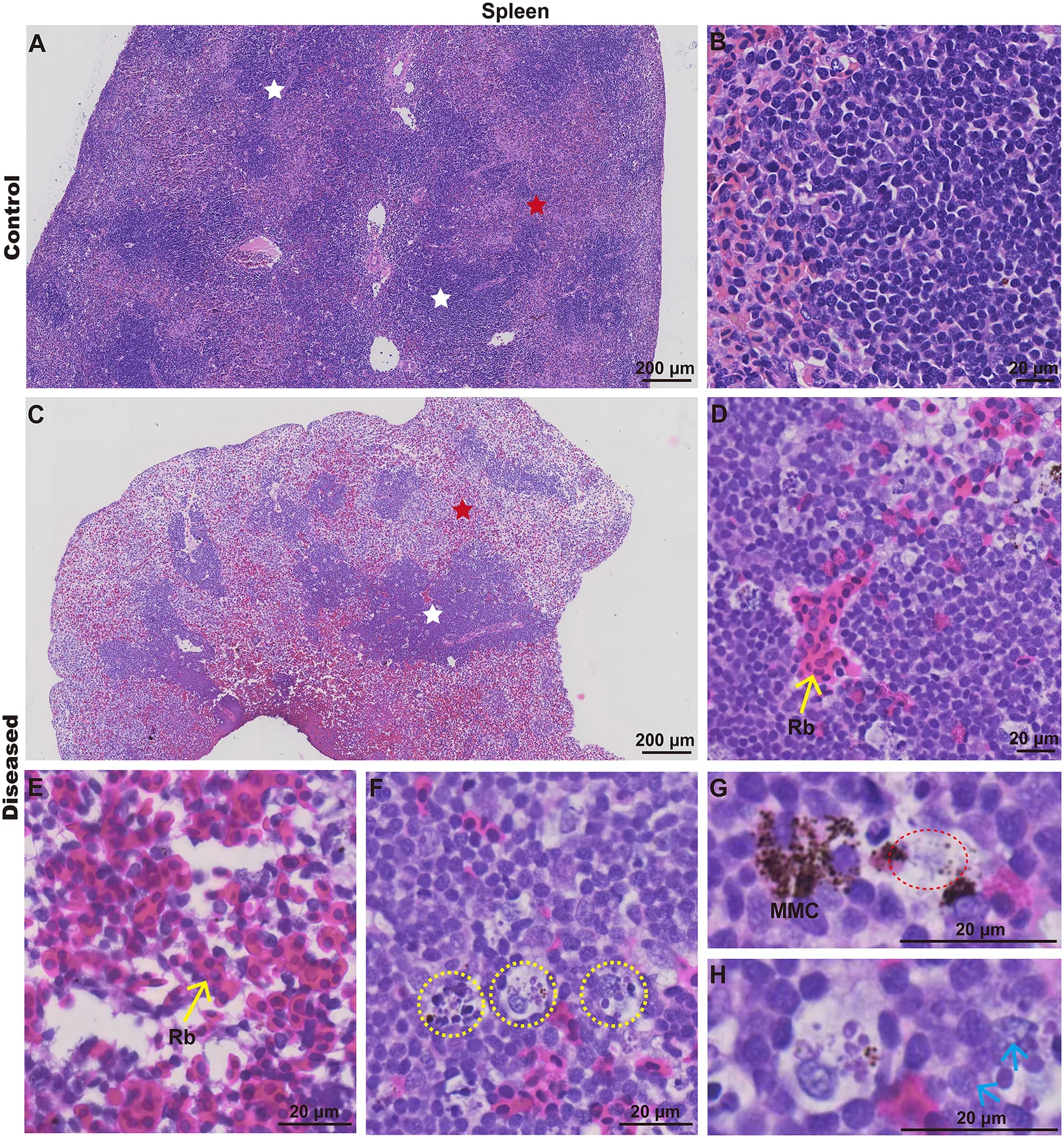
Histopathological examination of the spleen in hybrid sturgeons. **(A, B)** Control group with clear distinction between red pulp (red star) and white pulp (white star). **(C–E)** Diseased group showing obscured demarcation between pulps, white pulp atrophy, and extensive diffusion of red blood cells (Rb). **(F–H)** Diseased group exhibiting macrophages engulfing cellular debris (yellow dashed circles) and increased melano-macrophage centers (MMC); blue arrows indicate nuclear fragmentation and vacuolization.

**Figure 5:**
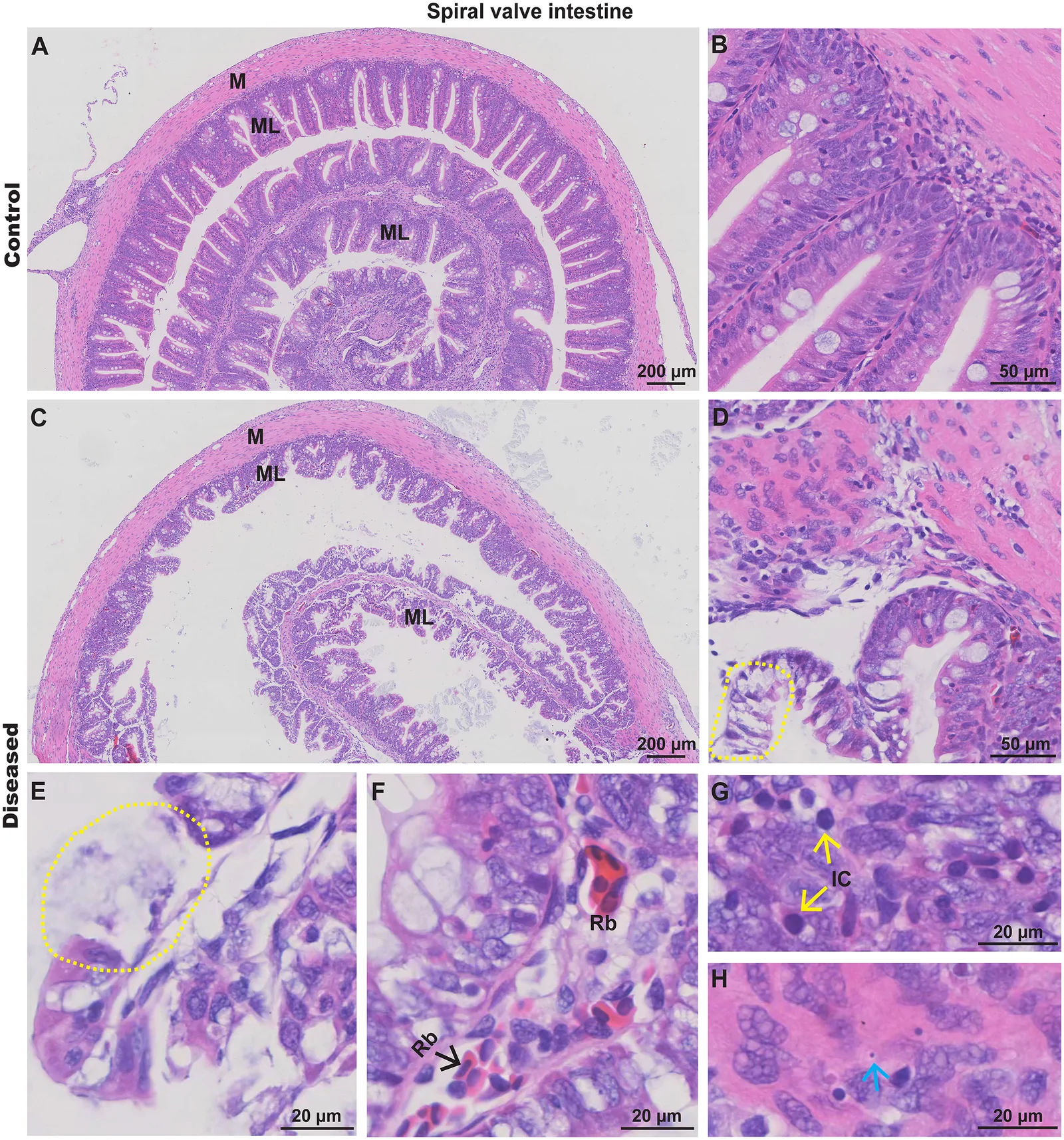
Histopathological examination of the spiral valve intestine in hybrid sturgeons. **(A, B)** Control group showing intact mucosa (M) and muscular layers (ML). **(C–E)** Diseased group showing atrophied mucosal folds and exfoliation of intestinal epithelial cells (yellow dashed circles). **(F–H)** Diseased group exhibiting congestion (Rb) and inflammatory cell (IC) infiltration in the lamina propria; blue arrows indicate necrotic nuclei.

**Figure 6:**
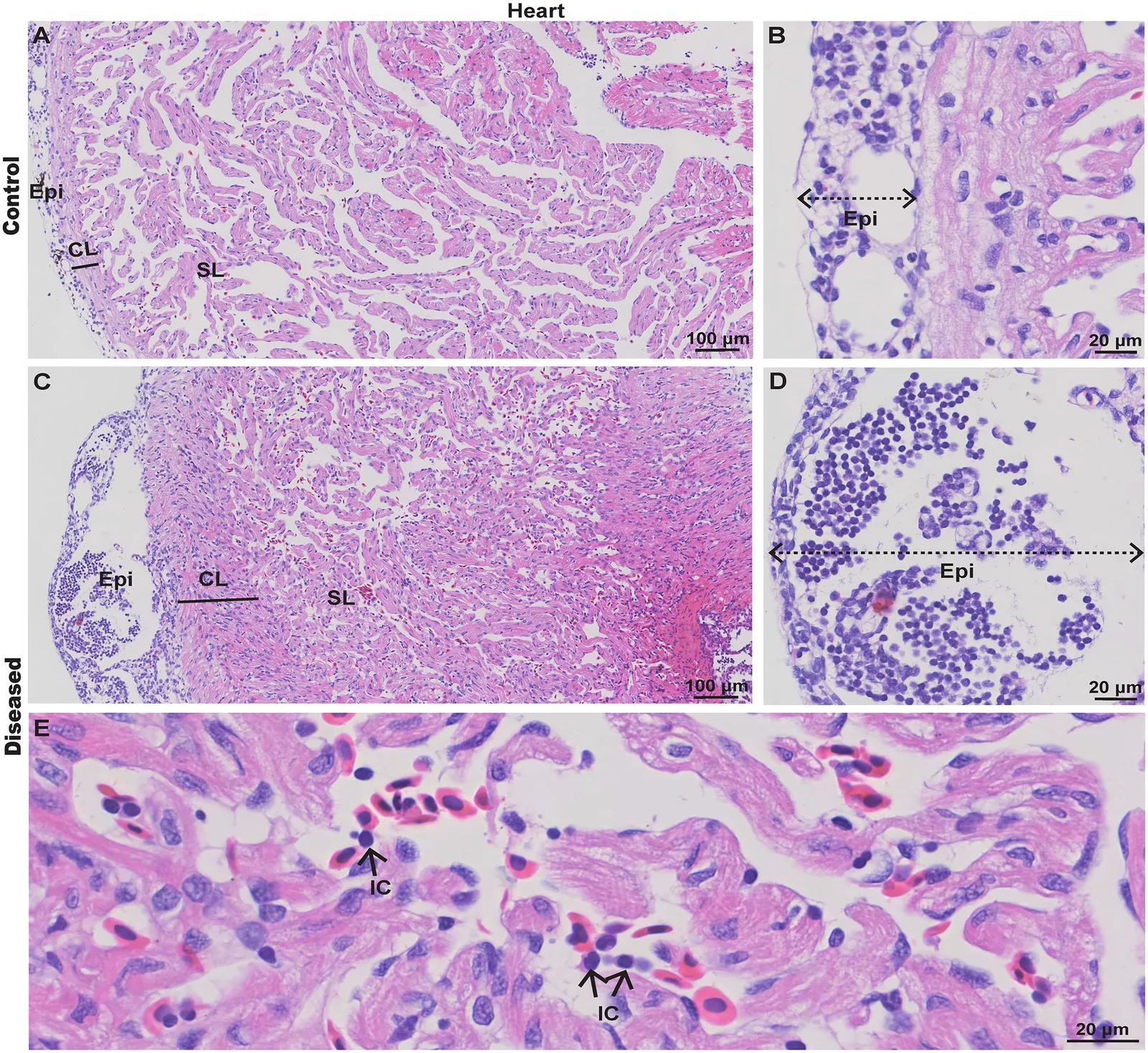
Histopathological examination of the heart in hybrid sturgeons. **(A, B)** Control group with normal epicardium (Epi), compact layer (CL), and spongy layer (SL). **(C, D)** Diseased group showing epicardial thickening with massive inflammatory accumulation (indicated by dashed lines). **(E)** Diseased group showing inflammatory cells (IC) infiltrating between myocardial fibers.

**Figure 7:**
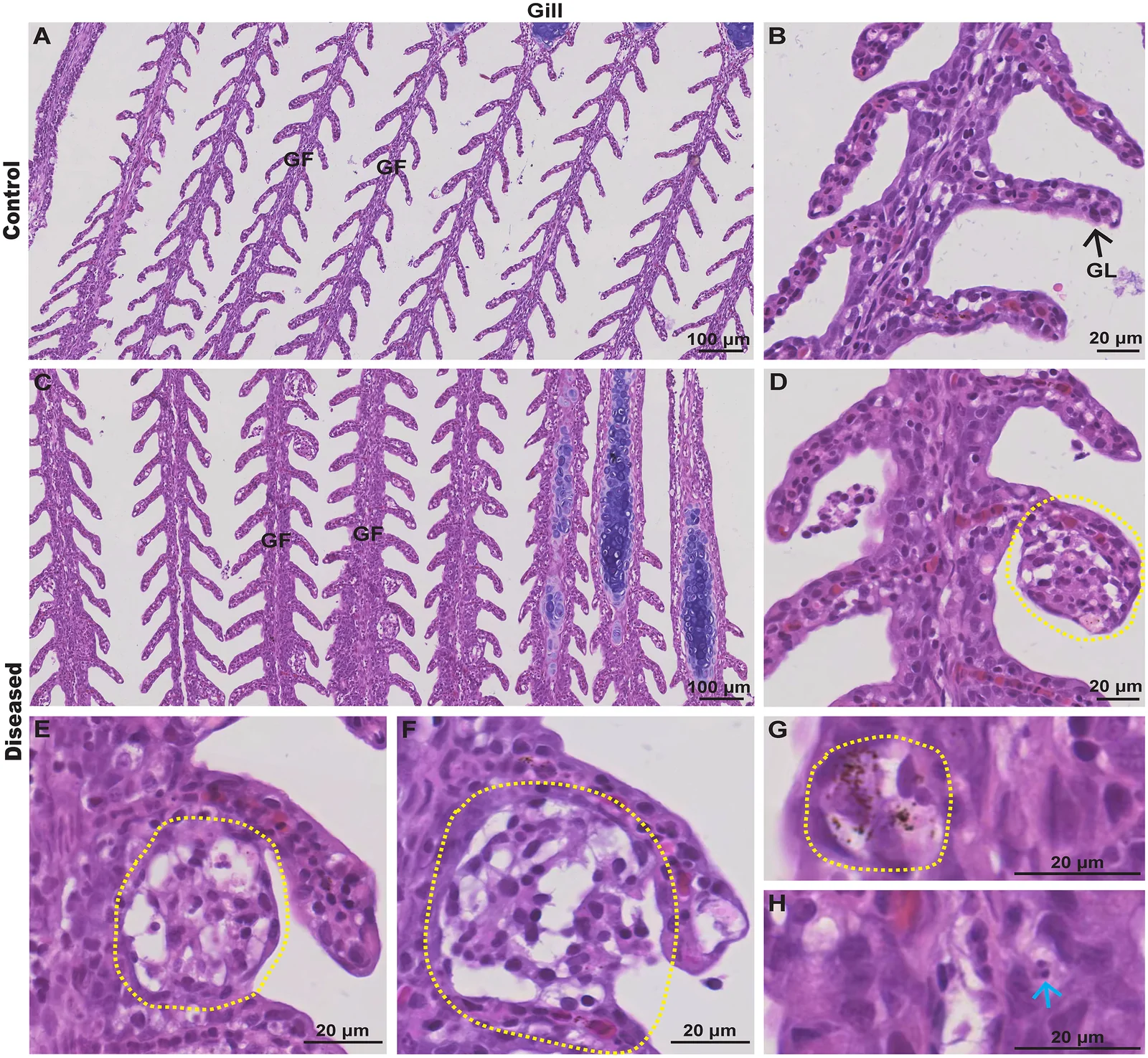
Histopathological examination of the gills in hybrid sturgeons. **(A, B)** Control group showing neatly arranged gill filaments (GF) and secondary lamellae (GL). **(C–F)** Diseased group exhibiting hypertrophied distal ends of lamellae due to hyperplasia and exudation (yellow dashed circles). **(G, H)** Diseased group showing localized recruitment of melanomacrophages (yellow dashed circles) and nuclear pyknosis (blue arrow).

Histopathological examination of multiple tissues from naturally diseased hybrid sturgeons revealed systemic damage and pronounced inflammatory responses. In the liver, the healthy control group displayed organized hepatic cord structures (**Figures 3A, B**). Conversely, the tissue architecture in diseased individuals was disrupted, characterized by vascular dilation and a massive accumulation of red blood cells within the blood vessels and hepatic sinusoids (**Figures 3C, D**). Significant perivascular infiltration of inflammatory cells was also observed (**Figure 3E**). Furthermore, hepatocytes exhibited severe vacuolar degeneration, accompanied by eosinophilic cytoplasm and focal necrotic areas where cellular boundaries became indistinct (**Figure 3F**). Bacterial clusters were identified within the hepatic parenchyma (red dashed circle, **Figure 3G**). Pathological alterations in hepatocyte nuclei were evident, including irregular morphologies and localized pyknosis (**Figure 3H**). In the spleen, the control group maintained a relatively clear distinction between red and white pulp, with cells arranged in a dense and uniform pattern (**Figures 4A, B**). In diseased sturgeons, this demarcation became obscured, and the white pulp regions showed marked atrophy. The tissue exhibited severe congestion, with red blood cells diffusing extensively throughout the splenic parenchyma (**Figures 4C–E**). Macrophages were observed engulfing substantial amounts of cellular debris (yellow dashed circles, **Figure 4F**). Melano-macrophage centers (MMCs) were identified within the tissue (**Figure 4G**), and specific regions demonstrated nuclear pyknosis, karyorrhexis, and cytoplasmic vacuolization (blue arrows, **Figure 4H**). Observations of the spiral valve intestine revealed that the control group possessed intact mucosal and muscular layers, with a regular distribution of goblet cells (**Figures 5A, B**). In the diseased group, however, the mucosal layer was damaged, and the intestinal folds exhibited significant atrophy and shedding, leading to the exfoliation of intestinal epithelial cells (**Figures 5C–E**). Marked congestion and the infiltration of inflammatory cells were noted within the submucosa and lamina propria (**Figures 5F, G**). Additionally, some epithelial cells displayed characteristic features of nuclear pyknosis or necrosis (**Figure 5H**). The heart of control sturgeons showed clear structures of the epicardium, compact layer, and spongy layer, with regularly arranged cells (**Figures 6A, B**). In contrast, the epicardium of diseased fish was thickened, and the myocardial fibers became disorganized, with a substantial accumulation of inflammatory cells and exudates within the tissue (**Figures 6C, D**). Inflammatory cells were also observed scattered between the myocardial fibers (**Figure 6E**). Finally, the gills of the control group presented neatly arranged gill filaments and slender, parallel secondary lamellae (**Figures 7A, B**). In the diseased fish, the distal ends of some lamellae became hypertrophic due to epithelial cell hyperplasia and inflammatory exudation (yellow dashed circles, **Figures 7C–F**). Localized recruitment of melano-macrophages was observed in damaged areas (**Figure 7G**), and certain cells exhibited nuclear pyknosis (**Figure 7H**).

### 3.5 Ultrastructural analysis of tissues

**Figure 8:**
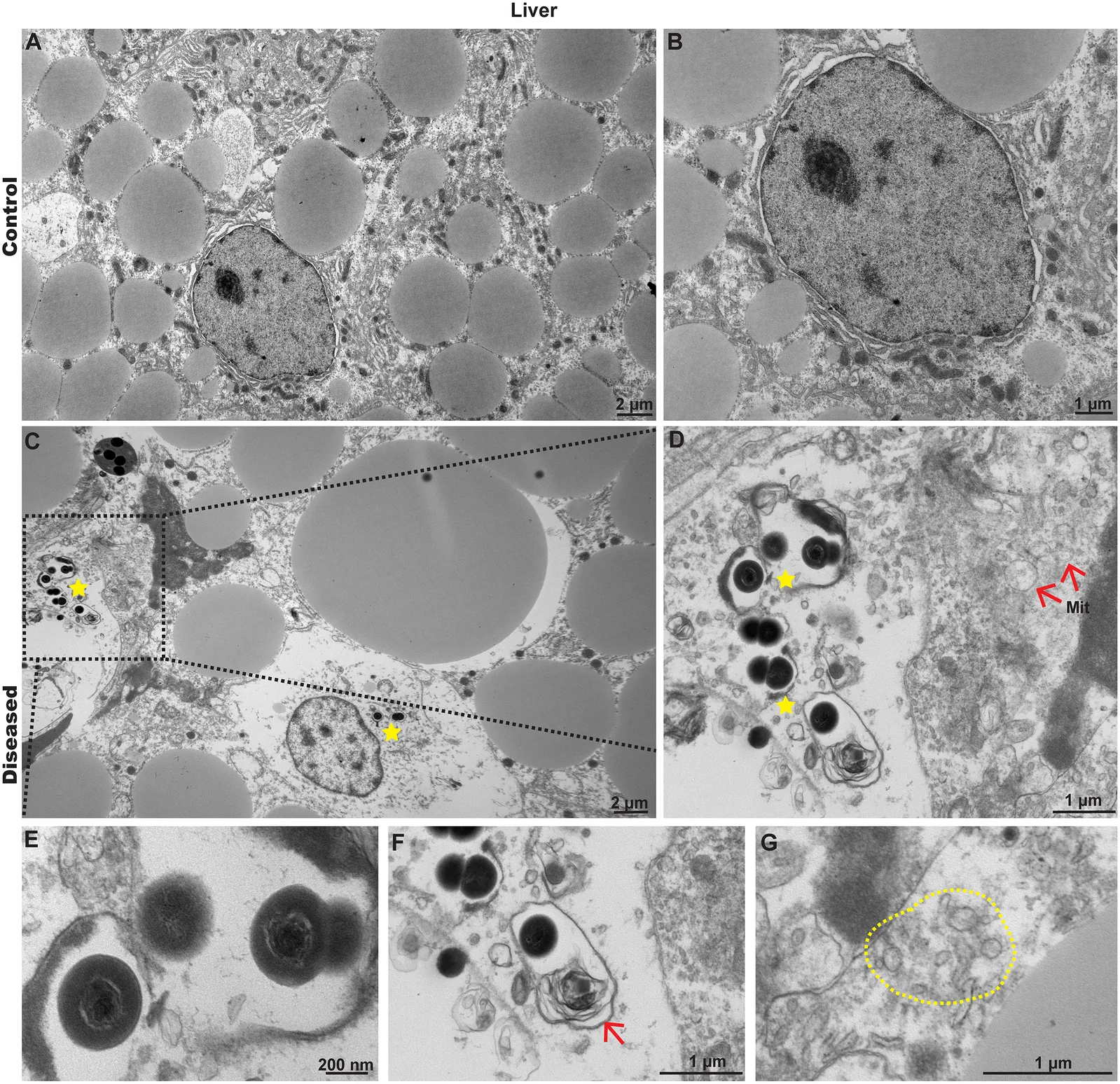
TEM observation of the liver in hybrid sturgeons. **(A, B)** Control group: Hepatocytes show intact ultrastructure with lipid droplets and normal nuclear morphology. **(C, D)** Diseased group: Clusters of bacteria (yellow stars) are localized within the damaged cytoplasm, and mitochondria (Mit) exhibit swelling and vacuolization. **(E)** Diseased group: Bacteria encapsulated by membrane structures undergoing binary fission, surrounded by organelle debris. **(F, G)** Diseased group: Presence of autophagosome-like structures, also resembling myelin-like bodies (red arrow), and extensive autophagy-like vacuolar structures (yellow dashed circle).

**Figure 9:**
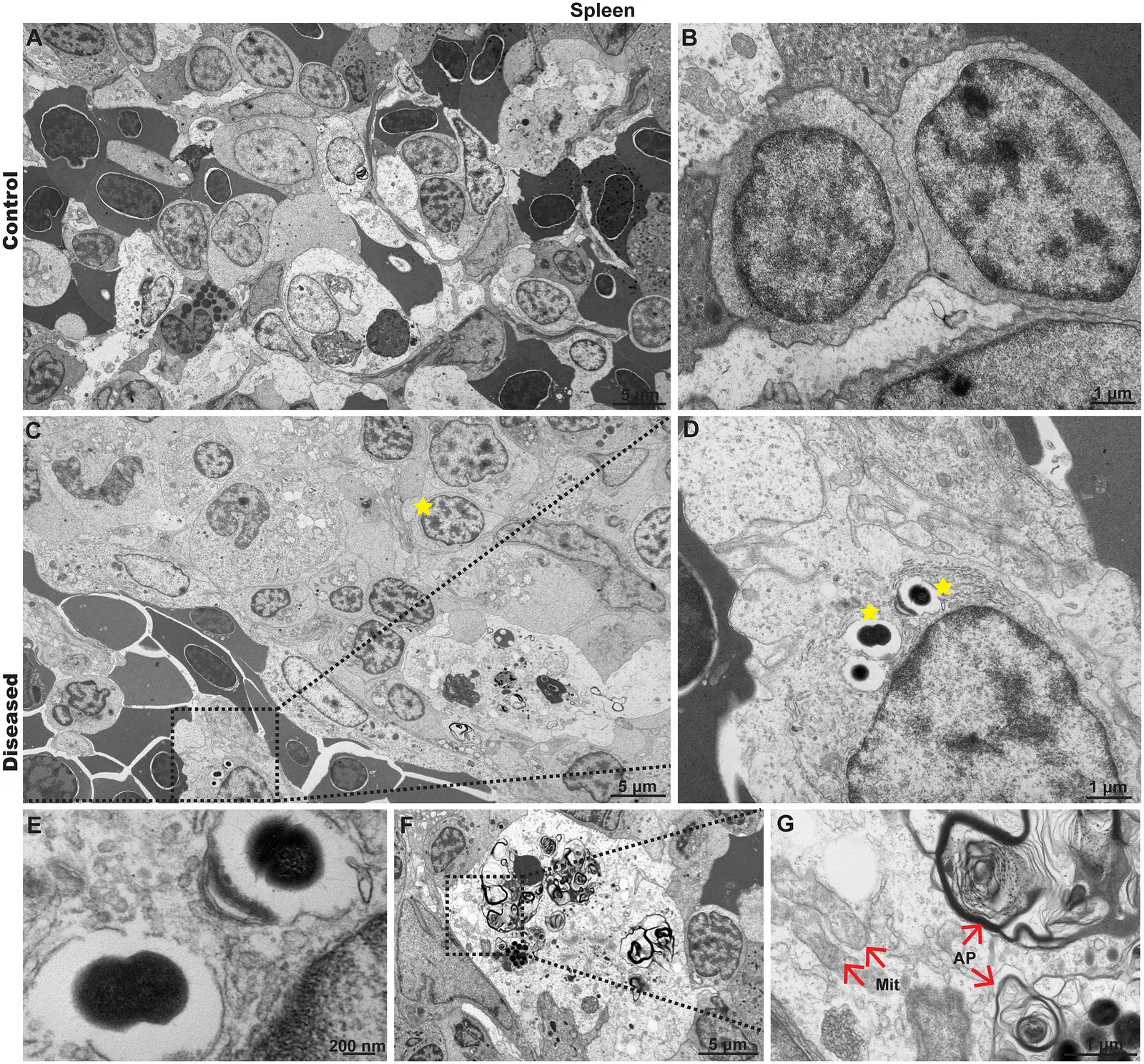
TEM observation of the spleen in hybrid sturgeons. **(A, B)** Control group: Immune cells are densely arranged with normal nuclear architecture. **(C)** Diseased group: Bacterial clusters (yellow stars) are distributed within the splenic parenchyma. **(D, E)** Diseased group: Pathogens within cytoplasmic vacuoles undergoing active division. **(F, G)** Diseased group: Numerous autophagy-related precursor-like structures (AP) and severely damaged/swollen mitochondria (Mit).

**Figure 10:**
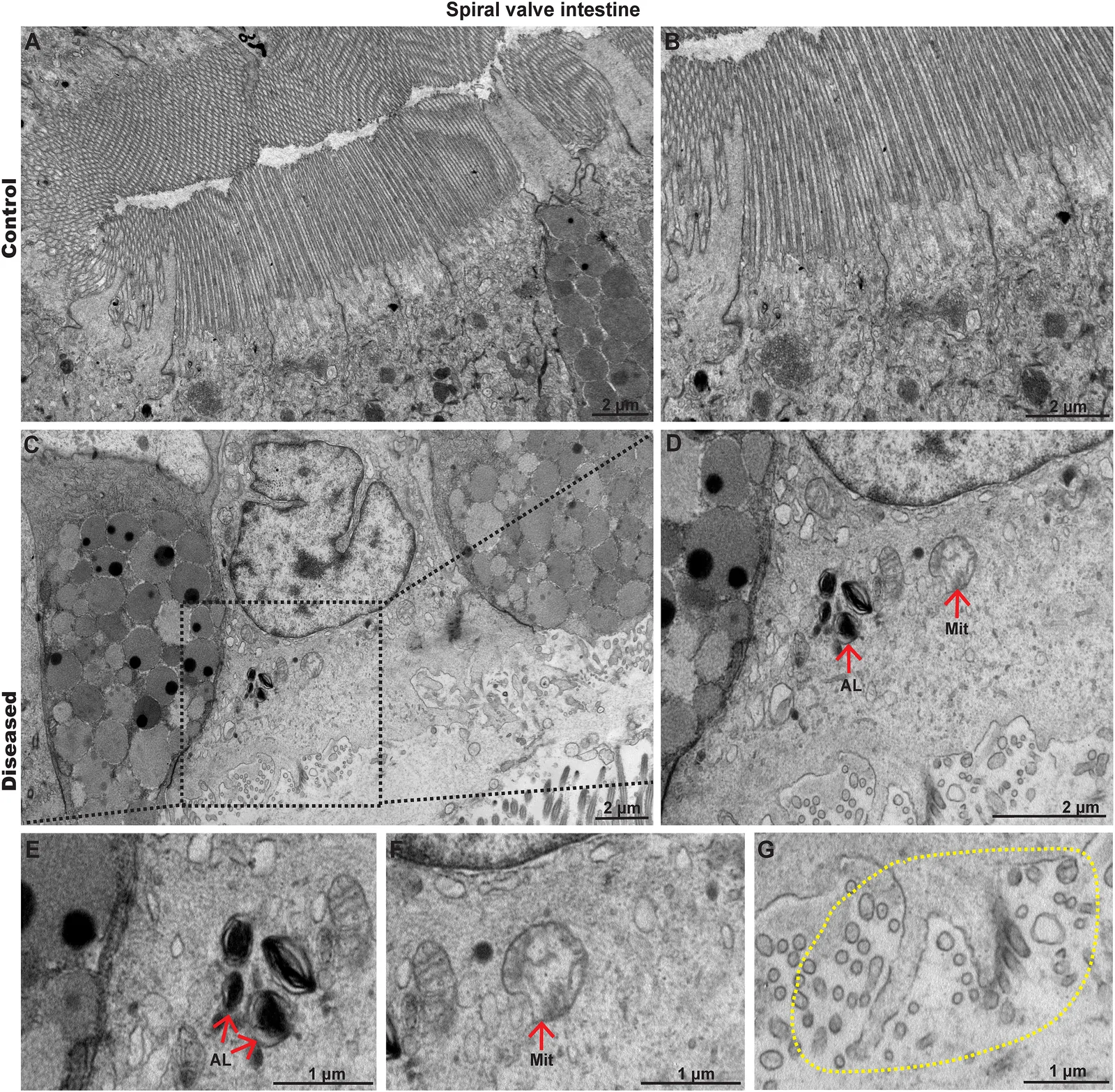
TEM observation of the spiral valve intestine in hybrid sturgeons. **(A, B)** Control group: Intestinal epithelial cells showing neatly arranged and dense microvilli. **(C–F)** Diseased group: Accumulation of electron-dense secondary lysosomes (AL) and severely swollen mitochondria (Mit) in the cytoplasm. **(G)** Diseased group: Fragmentation and shedding of microvilli (yellow dashed circle), indicating damage to the mucosal barrier.

**Figure 11:**
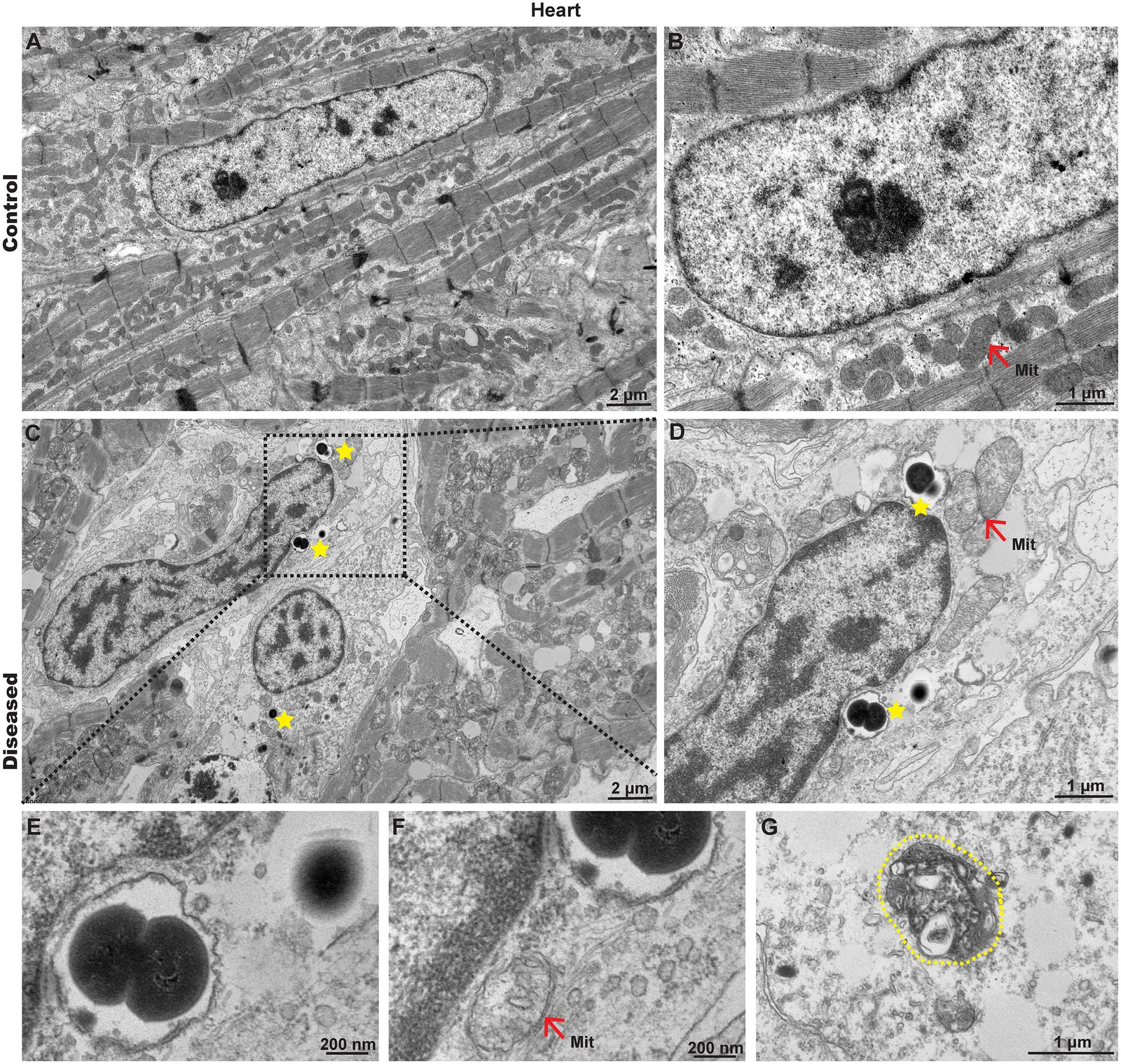
TEM observation of the heart in hybrid sturgeons. **(A, B)** Control group: Well-organized myocardial fibers with normal mitochondrial (Mit) structures. **(C)** Diseased group: Bacterial clusters (yellow stars) in the interstitial spaces with localized fiber lysis. **(D–F)** Diseased group: Bacterial binary fission and mitochondria (Mit) showing pronounced swelling and vacuolization. **(G)** Diseased group: Autophagosome-like structures (yellow dashed circle) within the cytoplasm.

**Figure 12:**
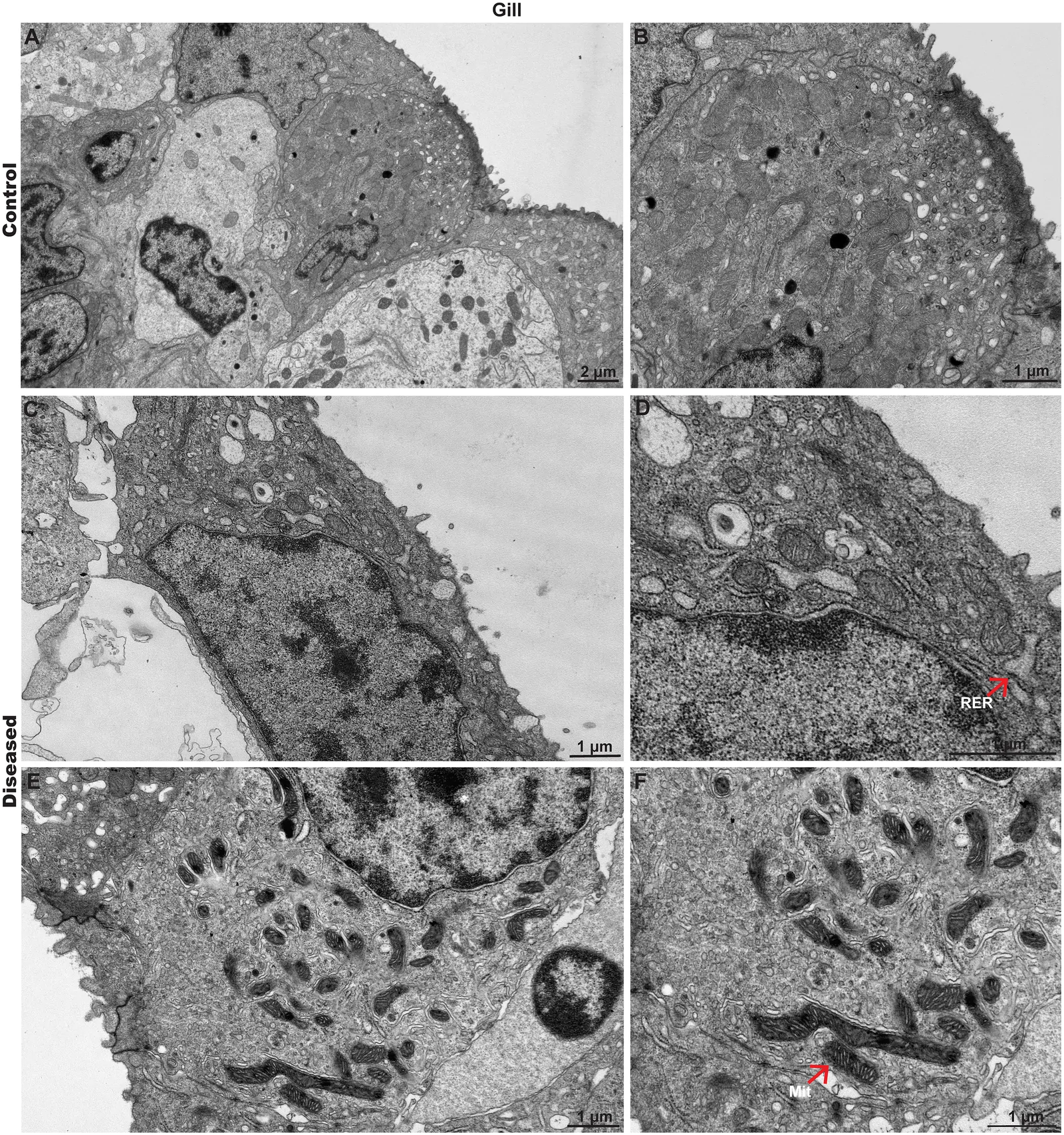
TEM observation of the gills in hybrid sturgeons. **(A, B)** Control group: Respiratory epithelial cells with intact ultrastructure and normal organelle distribution. **(C, D)** Diseased group: Epithelial cells showing widened perinuclear spaces and significant expansion of the rough endoplasmic reticulum (RER). **(E, F)** Diseased group: Highly swollen mitochondria (Mit) with a complete loss of cristae structures.

TEM was employed to evaluate the subcellular damage and pathogen distribution in key organs of naturally diseased hybrid sturgeons. In the liver, hepatocytes of the healthy control group displayed intact ultrastructure, regular nuclear morphology, and numerous lipid droplets within the cytoplasm (**Figures 8A, B**). Conversely, hepatocytes in the diseased group exhibited pathological alterations, characterized by clusters of bacteria (yellow stars) localized within the damaged cytoplasm. Mitochondria in these cells showed marked swelling, cristae lysis, and vacuolization (**Figures 8C, D**). High-magnification observations revealed that the bacteria were frequently encapsulated by membrane structures, with distinct signs of active binary fission accompanied by surrounding organelle debris (**Figure 8E**). Furthermore, autophagosome-like structures, also resembling myelin-like bodies (red arrow, **Figure 8F**), and extensive autophagy-like vacuolar structures (yellow dashed circle) were evident (**Figure 8G**). Ultrastructural analysis of the spleen showed that the control tissue was composed of various immune cells arranged in a dense, uniform pattern with normal nuclear architecture (**Figures 9A, B**). In diseased fish, bacterial clusters (yellow stars) were widely distributed throughout the splenic parenchyma (**Figure 9C**). Under higher magnification, pathogens within cytoplasmic vacuoles were observed undergoing active division (**Figures 9D, E**). The tissue also exhibited numerous autophagy-related precursor-like structures and severely swollen mitochondria (**Figures 9F, G**). In the spiral valve intestine, the control group possessed intestinal epithelial cells with neatly arranged and dense microvilli (**Figures 10A, B**). In contrast, the microvilli in the diseased group were fragmented or exfoliated, while the cytoplasm contained an accumulation of electron-dense secondary lysosomes and severely swollen mitochondria (**Figures 10C–F**). Magnified views confirmed the disruption and shedding of microvilli (yellow dashed circle), reflecting damage to the intestinal mucosal barrier (**Figure 10G**). The heart of control sturgeons displayed well-organized myocardial fibers with distinct striation patterns and normal mitochondrial structures (**Figures 11A, B**). In the diseased individuals, bacteria (yellow stars) were found scattered or clustered within the interstitial spaces of myocardial fibers, which exhibited localized fragmentation and lysis (**Figure 11C**). Typical bacterial binary fission was observed alongside mitochondria showing pronounced swelling and vacuolization (**Figures 11D–F**). Additionally, autophagosome-like structures (yellow dashed circle) were identified within the cytoplasm (**Figure 11G**). Finally, ultrastructural observation of the gills revealed that respiratory epithelial cells in the control group had intact structures and normal organelle distribution (**Figures 12A, B**). Diseased sturgeons, however, demonstrated epithelial cell degeneration, characterized by widened perinuclear spaces and significant expansion of the rough endoplasmic reticulum (**Figures 12C, D**). Mitochondria appeared swollen with a complete loss of cristae (**Figures 12E, F**). These subcellular changes further indicate severe systemic tissue damage in naturally diseased hybrid sturgeons.

### 3.6 PacBio SMRT sequencing, Illumina RNA-seq, and differential expression analysis

Mortality in the challenged group began on day 2 post-injection but remained limited at the 48-h sampling point. By this time, the challenged fish had developed clinical signs and gross abnormalities similar to those observed during the natural outbreak, including an empty and distended intestine, redness and swelling around the anus, and skin ulceration in some fish. No mortality or comparable clinical abnormalities were observed in the PBS-injected control group. At 48 h post-injection, bacteria were successfully re-isolated from the internal organs of all three challenged fish selected for transcriptome sequencing. The recovered isolates exhibited colony morphology consistent with that of the original challenge strain and were confirmed as *S. iniae* using the species-specific *lctO* PCR assay. These findings supported the successful establishment of infection in the challenged fish used for RNA-seq.

**Figure 13:**
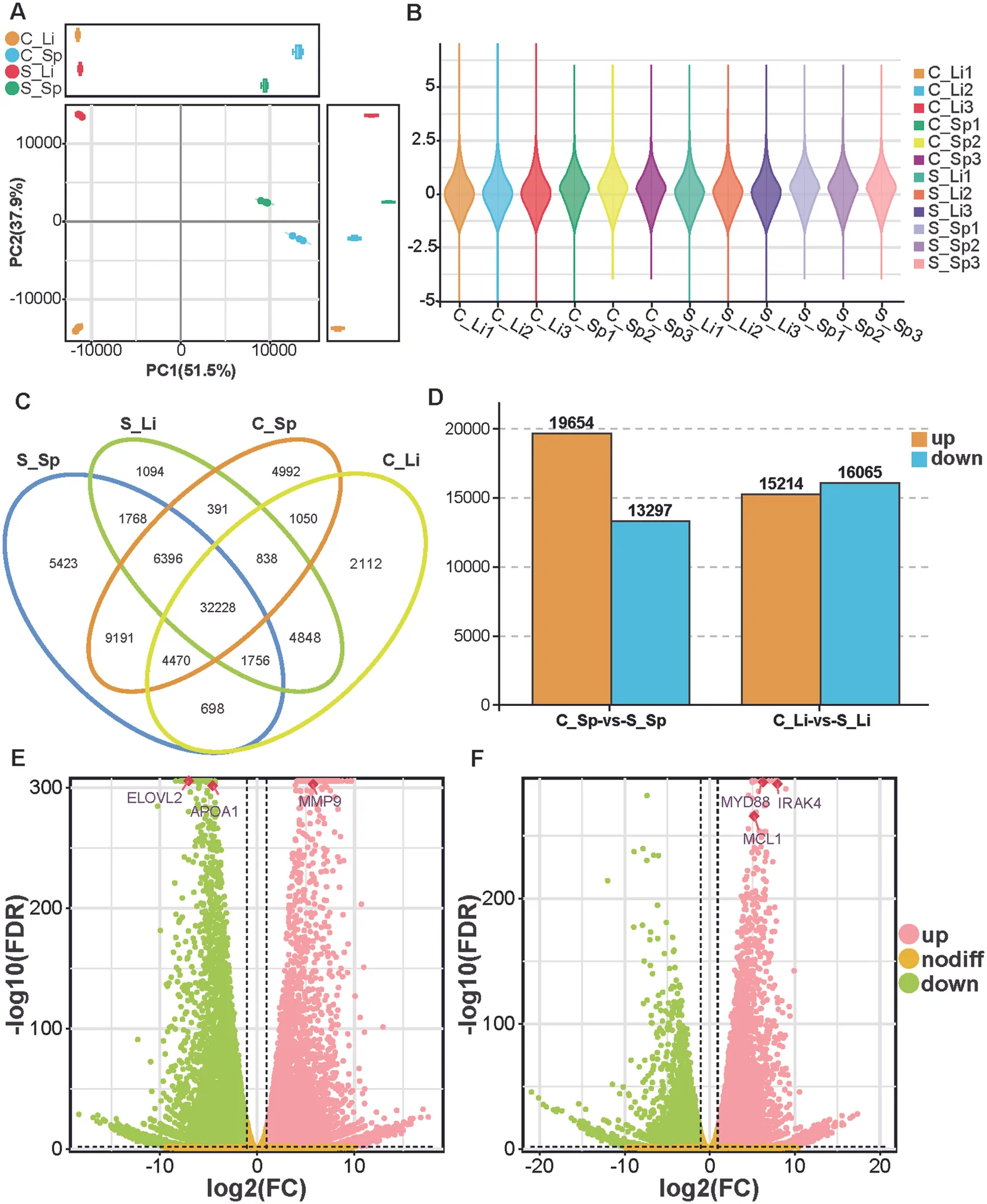
Transcriptomic profiles and differential transcript expression in the liver and spleen of hybrid sturgeon following *S. iniae* challenge. **(A)** Principal component analysis (PCA) of the 12 Illumina RNA-seq libraries. **(B)** Violin plot illustrating the distribution of transcript expression levels across the 12 transcriptome libraries. **(C)** Venn diagram showing the numbers of shared and uniquely expressed transcripts among the four tissue-treatment groups. **(D)** Numbers of significantly upregulated and downregulated transcripts in the spleen and liver following *S. iniae* challenge. **(E, F)** Volcano plots of DETs in the liver **(E)** and spleen **(F)**.

PacBio SMRT sequencing of the pooled RNA sample generated 5,375,337 HiFi reads, comprising 7.02 Gb of sequence data. The HiFi reads had a mean length of 1,306 bp and an N50 length of 1,434 bp. After primer identification and read classification, 5,351,335 full-length reads were obtained, of which 5,298,928 were full-length non-chimeric reads. Clustering generated 557,161 consensus isoforms. After redundancy removal, 120,215 non-redundant transcripts were retained, with a mean length of 1,732.54 bp and an N50 length of 2,001 bp. BUSCO analysis identified 94.4% of the expected complete orthologues, indicating high completeness of the transcript reference. For the 12 liver and spleen libraries included in the present analysis, Illumina sequencing generated 5.53-7.29 Gb of clean data per sample. The Q20 and Q30 rates ranged from 98.72% to 98.92% and from 95.01% to 95.65%, respectively, while the GC content ranged from 44.23% to 48.42% (**Supplementary Table 5**). Principal component analysis (PCA) was conducted to assess the global gene expression patterns, revealing a clear separation between experimental groups (**Figure 13A**). Violin plots further demonstrated that the gene expression distribution was uniform across all 12 samples (**Figure 13B**). A total of 32,228 transcripts were detected in all four groups. In addition, 2,112, 4,992, 1,094, and 5,423 transcripts were uniquely detected in the PBS control liver, PBS control spleen, challenged liver, and challenged spleen groups, respectively (**Figure 13C**). Using FDR < 0.05 and |log2 fold change| > 1 as the screening criteria, 31,279 differentially expressed transcripts were identified in the liver following *S. iniae* challenge, including 15,214 upregulated and 16,065 downregulated transcripts. In the spleen, 32,951 differentially expressed transcripts were detected, including 19,654 upregulated and 13,297 downregulated transcripts (**Figure 13D**). Representative expression changes included the upregulation of *MMP9* and downregulation of *ELOVL2* and *APOA1* in the liver, together with the upregulation of *MYD88*, *IRAK4*, and *MCL1* in the spleen (**Figures 13E, F**).

### 3.7 GO and KEGG enrichment analysis

**Figure 14:**
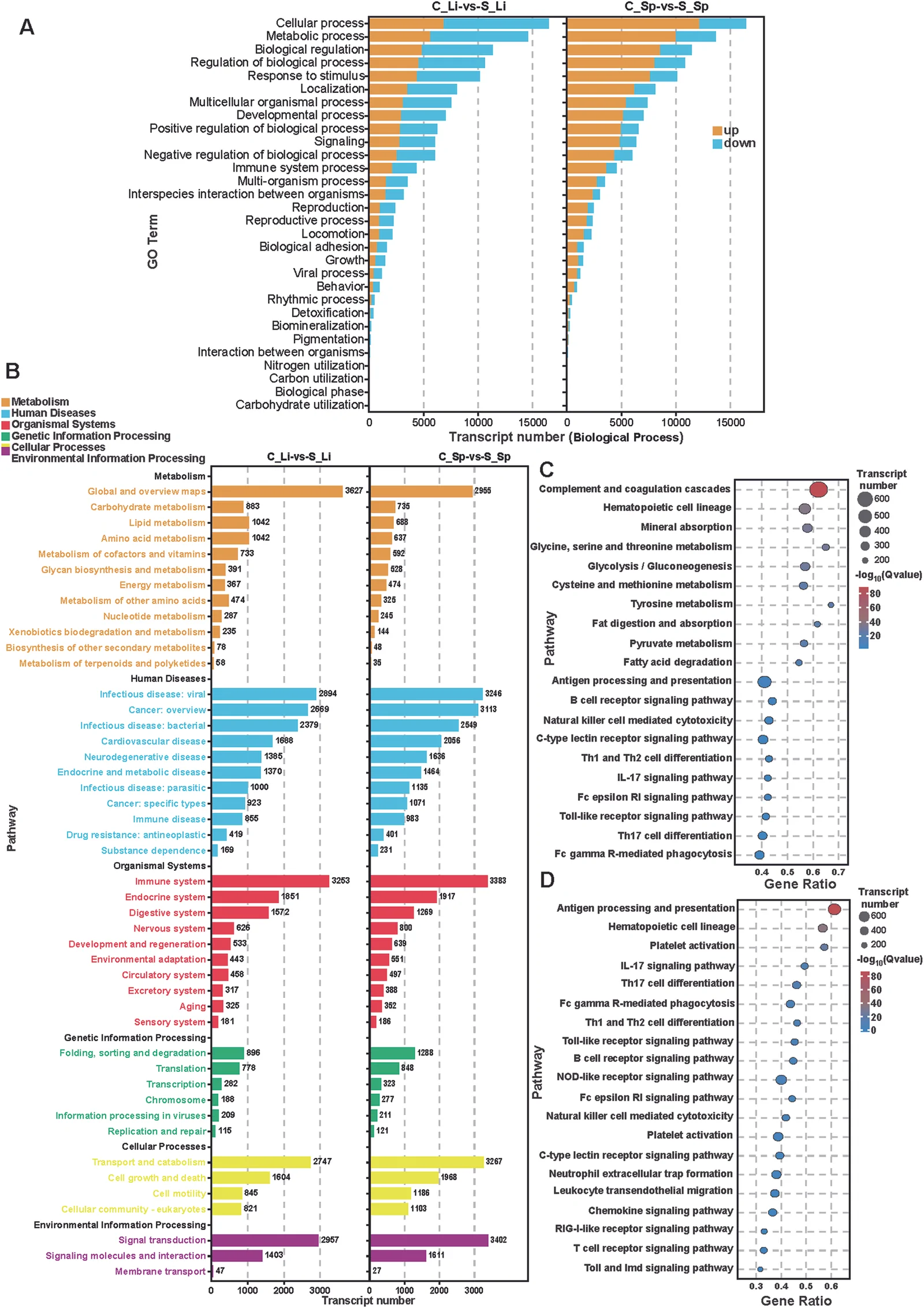
GO and KEGG functional classification and enrichment analyses of DETs in the liver and spleen following *S. iniae* challenge. **(A)** GO enrichment bar plots showing the Biological Process (BP) classification of DETs in the liver and spleen. **(B)** KEGG pathway classification of DETs across major categories. **(C, D)** Bubble plots of significantly enriched KEGG pathways in the liver **(C)** and spleen **(D)**.

GO analysis was used to examine the biological functions associated with the DETs in the liver and spleen following *S. iniae* challenge. Within the Biological Process (BP) category, transcripts from both tissues were mainly assigned to cellular process, metabolic process, biological regulation, regulation of biological process, response to stimulus, localization, and multicellular organismal process. Transcripts related to signaling and immune system process were also found in both tissues (**Figure 14A**). KEGG classification distributed the differentially expressed transcripts across six broad categories: Metabolism, Human Diseases, Organismal Systems, Genetic Information Processing, Cellular Processes, and Environmental Information Processing. Among these categories, global and overview maps contained the largest number of metabolism-related transcripts. Immune system and signal transduction were also prominent subcategories within Organismal Systems and Environmental Information Processing, respectively (**Figure 14B**). The KEGG enrichment profiles differed between the liver and spleen. In the liver, enriched pathways covered both metabolic and immune-related functions. These included complement and coagulation cascades, hematopoietic cell lineage, glycine, serine and threonine metabolism, glycolysis/gluconeogenesis, cysteine and methionine metabolism, tyrosine metabolism, fat digestion and absorption, pyruvate metabolism, and fatty acid degradation. Several immune pathways were also enriched, including antigen processing and presentation, B cell receptor signaling, natural killer cell-mediated cytotoxicity, C-type lectin receptor signaling, IL-17 signaling, Toll-like receptor signaling, and Fc gamma R-mediated phagocytosis (**Figure 14C**). In the spleen, most of the enriched pathways were related to immune functions. The main pathways included antigen processing and presentation, hematopoietic cell lineage, IL-17 signaling, Th17 cell differentiation, Fc gamma R-mediated phagocytosis, Th1 and Th2 cell differentiation, Toll-like receptor signaling, B cell receptor signaling, NOD-like receptor signaling, natural killer cell-mediated cytotoxicity, C-type lectin receptor signaling, and Toll and Imd signaling (**Figure 14D**).

### 3.8 GSEA of core pathways

**Figure 15:**
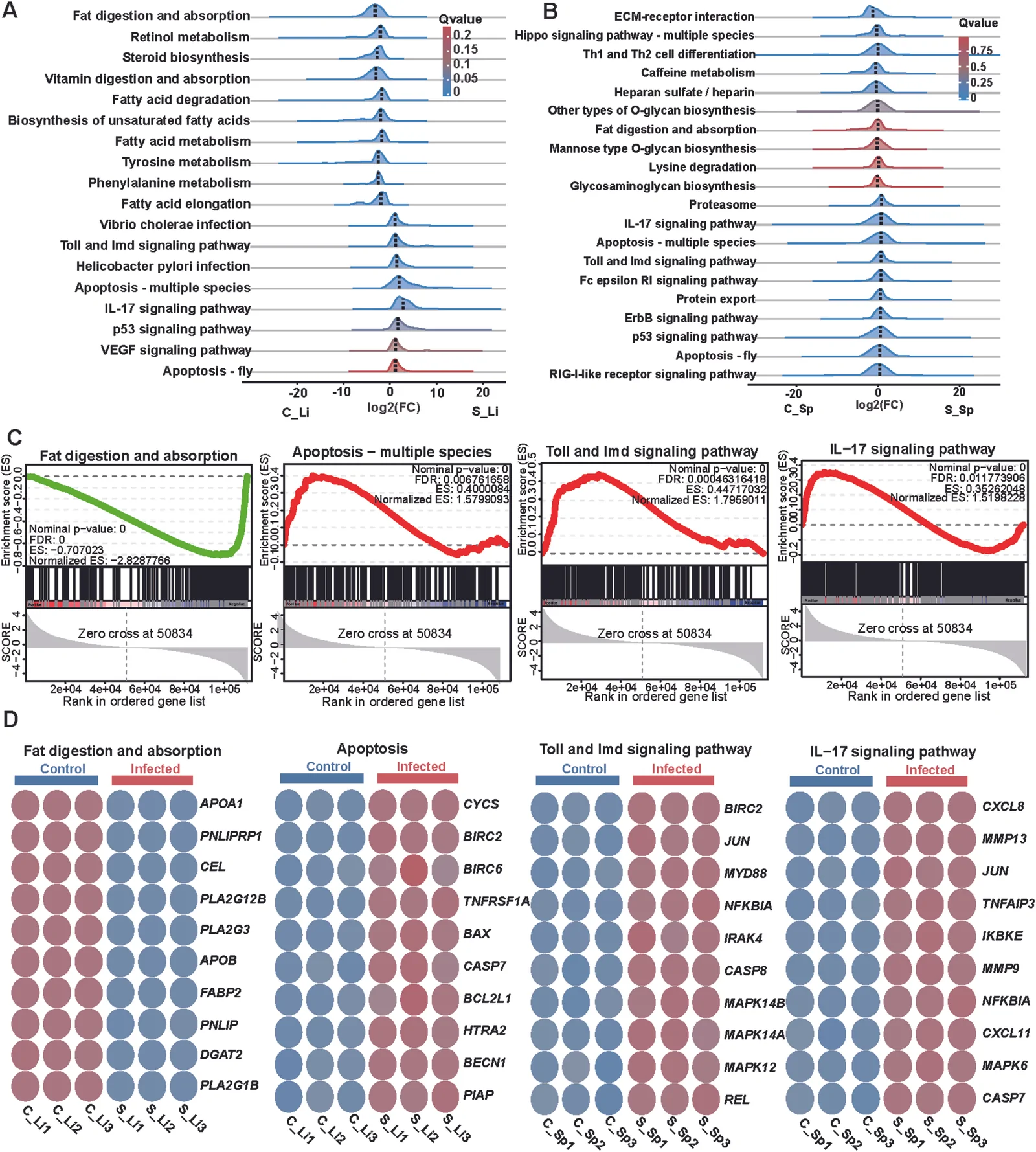
Gene set enrichment analysis and expression patterns of representative pathway-related transcripts in the liver and spleen following *S. iniae* challenge. **(A–B)** Ridge plots displaying the distribution of the top 20 enriched pathways in the liver **(A)** and spleen **(B)**. **(C)** GSEA enrichment plots for the fat digestion and absorption and apoptosis-multiple species pathways in the liver, and the Toll and Imd and IL-17 signaling pathways in the spleen. The nominal *P*-value, false discovery rate (FDR), enrichment score (ES), and normalized enrichment score (NES) are shown in each plot. **(D)** Heatmaps of core transcripts expression levels within the corresponding pathways.

GSEA was performed to identify coordinated pathway-level changes following *S. iniae* challenge. In the liver, the analysis highlighted several pathways associated with lipid and nutrient metabolism, including fat digestion and absorption, retinol metabolism, steroid biosynthesis, vitamin digestion and absorption, fatty acid degradation, biosynthesis of unsaturated fatty acids, and fatty acid metabolism. Immune- and cell death-related pathways, such as Toll and Imd signaling, IL-17 signaling, p53 signaling, and apoptosis, were also detected (**Figure 15A**). In the spleen, the GSEA profiles included Th1 and Th2 cell differentiation, IL-17 signaling, Toll and Imd signaling, RIG-I-like receptor signaling, apoptosis, Fc epsilon RI signaling, and several pathways related to extracellular matrix interactions and protein metabolism (**Figure 15B**). Four representative pathways were examined in greater detail. In the liver, fat digestion and absorption showed negative enrichment following challenge, whereas apoptosis - multiple species was positively enriched. In the spleen, both Toll and Imd signaling and IL-17 signaling showed positive enrichment (**Figure 15C**). The expression patterns of representative transcripts were consistent with the GSEA profiles. In the liver, transcripts associated with fat digestion and absorption, including *APOA1*, *PNLIPRP1*, *CEL*, *PLA2G12B*, *PLA2G3*, *APOB*, *FABP2*, *PNLIP*, *DGAT2*, and *PLA2G1B*, generally showed lower expression in the challenged group. By contrast, apoptosis-related transcripts, including *CYCS*, *BIRC2*, *BIRC6*, *TNFRSF1A*, *BAX*, *CASP7*, *BCL2L1*, *HTRA2*, and *BECN1*, generally showed higher expression after challenge. In the spleen, transcripts contributing to Toll and Imd signaling, such as *JUN*, *MYD88*, *NFKBIA*, *IRAK4*, *CASP8*, *MAPK14A*, *MAPK14B*, *MAPK12*, and *REL*, were more highly expressed in the challenged fish. A similar pattern was observed for IL-17 signaling transcripts, including *CXCL8*, *MMP13*, *JUN*, *TNFAIP3*, *IKBKE*, *MMP9*, *NFKBIA*, *CXCL11*, *MAPK6*, and *CASP7* (**Figure 15D**).

### 3.9 qPCR validation of RNA-seq results

Eight DETs were selected for RT-qPCR validation. NormFinder identified *β-actin* as the more stable reference gene, and it was therefore used for relative expression normalization. In the liver, RNA-seq showed that *FTH1*, *GPX1*, *PRDX5*, *DRAM1*, and *MMP13* were upregulated following *S. iniae* challenge, whereas *CD74*, *C1QC*, and *FABP7* were downregulated. The RT-qPCR results showed the same directions of change. In the spleen, *FTH1*, *GPX1*, *PRDX5*, *DRAM1*, *MMP13*, and *FABP7* were upregulated, while *CD74* and *C1QC* were downregulated in both the RNA-seq and RT-qPCR datasets. Although the fold-change values differed to some extent between the two methods, all eight transcripts showed consistent expression trends in both tissues (**Supplementary Figure 1**).

## 4 Discussion

Sturgeon farming has undergone substantial expansion driven by the escalating ecological and commercial significance of these species, despite some of their status as vulnerable or critically endangered (28, 29). However, this growth faces formidable challenges arising from the intensification of aquaculture practices and the compounding stressors of global climate warming (30, 31). *Streptococcus* species are Gram-positive bacteria with high taxonomic diversity that infect a wide range of marine and freshwater fish. Warm-water fish species are particularly susceptible to streptococcosis, which has emerged as a globally prevalent and recalcitrant disease significantly threatening aquaculture health. Epidemiological data indicate that the primary pathogenic species include *S. iniae*, *S. agalactiae*, *S. parauberis*, and *S. dysgalactiae*, among which *S. iniae* is one of the most widely reported pathogens (32, 33). To date, *S. iniae* has been identified in various species, such as Japanese flounder (*Paralichthys olivaceus*) (34), red seabream (*Pagrus pagrus*) (35), Nile tilapia (*O. niloticus*) (7), golden pompano (*T. ovatus*) (8), and zebrafish (*Danio rerio*) (36), with clinical manifestations typically characterized by systemic septicemia and pericarditis. Notably, *S. iniae* is a zoonotic pathogen capable of causing human sepsis and meningitis, posing a potential risk to public health (37). In recent years, there have been increasing reports of *S. iniae* infections in sturgeons across various aquaculture regions, such as Siberian sturgeon (*A. baerii*) (12), Adriatic Sturgeon (*Acipenser naccarii*) (38), and farmed Chinese sturgeon (*A. sinensis*) (13), resulting in substantial economic losses (39). In the present study, a pathogenic strain was isolated from diseased hybrid sturgeons in Sichuan Province, which exhibited clinical manifestations primarily characterized by enteritis and systemic hemorrhaging. Phenotypic characterization and molecular biological analysis confirmed the isolate as *S. iniae*. These findings underscore the escalating threat posed by *S. iniae* to the sturgeon farming industry in China and highlight the urgent need for heightened surveillance and intensified research into effective mitigation strategies.

Regarding the identification of *S. iniae*, traditional bacteriological methods, such as physio-biochemical assays, often fail to provide accurate results. Furthermore, commercial biochemical identification kits frequently misidentify *S. iniae* as *S. uberis* or *S. dysgalactiae* (40). Therefore, to ensure the accuracy and reliability of the diagnosis, the present study employed a multidimensional identification strategy combining molecular biology and ultrastructural morphology. The *16S rRNA* gene sequence placed the isolate within the *S. iniae* clade, while the species-specific *lctO* PCR assay and subsequent sequence analysis provided more targeted evidence for its identity. Additionally, SEM and TEM observations further supported that the isolate possessed typical morphological characteristics of the genus *Streptococcus*, such as a spherical appearance and arrangement in pairs or long chains. By integrating morphological features, physicochemical properties, molecular identification, and typical pathological lesions, the etiological agent responsible for the mass mortality of farmed hybrid sturgeon in Sichuan Province was identified as *S. iniae*.

Clinical manifestations of *S. iniae* infection can be variable across different host species of fish. Previous reports of *S. iniae* infection in sturgeons and other fish species have described a range of clinical and pathological manifestations, most commonly including systemic septicemia, hemorrhage, hepatosplenic lesions, enteritis, meningitis, pericarditis, exophthalmia, and ocular opacity (12, 37, 41, 42). In sturgeons, *S. iniae*-associated outbreaks have been reported to cause systemic hemorrhage, hepatic and splenic lesions, and intestinal inflammation (12, 43, 44). In the present outbreak, the clinical signs and gross lesions observed in diseased hybrid sturgeons included lethargy, erratic swimming, pronounced anal redness and swelling, splenomegaly, hepatomegaly with hemorrhage, and severe abdominal distension. The most prominent gross pathological feature was the accumulation of numerous turbid gas bubbles and fluid within the spiral valve intestine and swim bladder. This gross lesion was further supported by histopathological evidence of extensive mucosal fold atrophy and epithelial desquamation in the spiral valve intestine. Compared with previous *S. iniae* reports, severe gas and fluid accumulation in the spiral valve intestine appears to be rarely described and may represent a notable pathological feature of the present outbreak. Conversely, ocular lesions, such as exophthalmia or unilateral ocular opacity, which have been reported in some *S. iniae* infections, were absent in the current cases (12, 45). Whether these divergent pathological manifestations in hybrid sturgeons following *S. iniae* infection are attributable to host-specific responses, strain-specific virulence factors, or environmental stressors warrants further investigation.

Antimicrobial susceptibility can vary considerably among *S. iniae* isolates. Based on the interpretive criteria used in this study, the present isolate was classified as susceptible to ampicillin, cefoperazone, ceftriaxone, and florfenicol. This pattern was broadly consistent with that reported for an *S. iniae* isolate from Siberian sturgeon *A. baerii* (12). In contrast, the present isolate was classified as resistant to both tested quinolones, enrofloxacin and levofloxacin, whereas the Siberian sturgeon isolate examined by Deng et al. was susceptible to quinolones (12). The responses to aminoglycosides were also variable: the isolate was susceptible to streptomycin, kanamycin, and tobramycin, showed an intermediate response to gentamicin, and was resistant to spectinomycin and amikacin. Variation in antimicrobial susceptibility has previously been reported among sturgeon-associated *S. iniae* isolates in Sichuan Province (39). Such differences may be related to strain background or previous antimicrobial exposure, although neither resistance genes nor antimicrobial-use history was examined in the present study. It should also be noted that these classifications were based on *in vitro* inhibition-zone measurements and do not directly demonstrate therapeutic efficacy in infected hybrid sturgeon. Even so, isolate-specific susceptibility testing remains useful when antimicrobial treatment is being considered, particularly when combined with prudent antimicrobial-use practices.

With respect to histopathological observations, the diseased hybrid sturgeon exhibited characteristic lesions including hepatic vasodilation, splenic white pulp atrophy with red pulp congestion, epicarditis, and branchial lamellar epithelial hyperplasia. These findings are generally consistent with previous reports in *T. ovatus* (8), *S. argus* (46) and *A. baerii* (12), supporting the presence of a common pathological pattern of systemic hemorrhagic septicemia associated with *S. iniae* infection. Notably, however, the hybrid sturgeon in this study displayed more profound gastrointestinal damage, specifically characterized by the atrophy of mucosal folds and desquamation of epithelial cells in the spiral valve intestine. This severe disruption of the mucosal barrier corresponds to the clinically observed intestinal gas accumulation and abdominal distension. Such remarkable intestinal lesions contrast with certain studies on teleost species where natural *S. iniae* infection elicited only mild enteritis (42). Nonetheless, prior studies have noted that low lethal doses of *S. iniae* can similarly induce conspicuous enteritis in Siberian sturgeon (*A. baerii*), manifested as severe intestinal lesions, inflammatory cell infiltration, submucosal edema, and epithelial necrosis and shedding (43). Furthermore, in acute infection studies of four-finger threadfin (*Eleutheronema tetradactylum*), *S. iniae* invasion not only led to significant differences in intestinal microbiota richness and diversity between healthy and infected fish but also caused severe structural disruptions to the intestinal villi architecture (44). Regarding the ultrastructural findings, spherical bacterial cells arranged individually, in pairs, or in clusters were observed within the parenchyma or interstitial spaces of the liver, spleen, and heart. Some bacterial cells also displayed morphological features consistent with binary fission. TEM alone could not establish their species identity; however, these observations provided additional morphological evidence. This morphology highly resembles the spherical cocci observed in the kidneys of infected Siberian sturgeon (47). Concurrently, the affected host cells exhibited pronounced mitochondrial swelling, cristae fragmentation, and the formation of numerous autophagy-like vacuolar structures. These cellular alterations suggest severe subcellular injury in the visceral organs of naturally diseased hybrid sturgeons. However, TEM observations alone cannot confirm the activation of autophagy or programmed cell death pathways. Therefore, these ultrastructural changes should be interpreted as morphological evidence of cellular injury, and their relationship with autophagy- or cell death-related pathways requires further experimental validation.

RNA-Seq has emerged as a useful approach for characterizing transcriptional changes associated with host-pathogen interactions (48). In the present study, the PacBio-derived full-length transcript set provided a transcript-level reference for Illumina RNA-seq, enabling expression to be quantified independently across biological replicates. The combined analysis revealed distinct but partly overlapping tissue-specific responses to *S. iniae* challenge. Whereas the liver exhibited changes involving both metabolic and immune-related processes, the splenic response was more strongly associated with immune functions. Similar immune-related transcriptional responses have been reported in other farmed fish following bacterial exposure (49, 50), suggesting that some components of the antibacterial response may be conserved across fish species.

The liver showed a clear combination of metabolic and cell death-related transcriptional changes after *S. iniae* challenge. The negative enrichment of the fat digestion and absorption pathway was accompanied by lower expression of transcripts such as *APOA1 and APOB*, suggests transcriptional reorganization of hepatic lipid metabolism at 48 h post-injection. Because the transcriptomic analysis was performed in the liver, these findings should not be interpreted as direct evidence of impaired intestinal lipid absorption. Metabolic adjustments accompanying immune and stress responses have also been reported in fish following bacterial infection, suggesting that immune–metabolic reprogramming may be an important component of the hepatic response (51, 52). Concurrent changes in pro- and anti-apoptotic transcripts further suggest dynamic regulation of cell death-related processes. The mitochondrial damage observed by TEM in naturally diseased fish provides complementary morphological evidence of cellular injury; however, because the transcriptomic and ultrastructural observations were obtained from different sets of fish, a direct mechanistic relationship between them cannot be established.

The spleen exhibited a predominantly immune-related transcriptional response following *S. iniae* challenge. Enrichment of Toll-like receptor-, NF-κB-, MAPK-, and IL-17-related signaling, together with altered expression of representative immune mediators such as *MYD88*, *IRAK4*, and *MMP13*, suggests coordinated regulation of innate inflammatory responses. MyD88 plays an important role in teleost Toll-like receptor signaling, and altered MyD88 expression has also been reported in black rockfish following *S. iniae* challenge (53). Similarly, IL-17 and its receptor family members have been identified in teleosts and shown to respond to bacterial infection (54). Collectively, these findings suggest that the spleen may be an important site of immune signaling during the early response to *S. iniae*, although the activity of these pathways and the functional roles of individual transcripts require further experimental validation.

Previous studies in sturgeons provide a useful context for interpreting these immune-related transcriptional changes. In Siberian sturgeon, experimental *S. iniae* infection induced time-dependent changes in the intestinal expression of the pro-inflammatory cytokines *tnf* and *il1β*, together with changes in mucin secretion and *muc2* expression (54). The expression of an NLRC3-like pattern-recognition receptor was also altered in the valvular intestine, duodenum and spleen during different stages of *S. iniae* infection (55). These findings suggest that the sturgeon response to *S. iniae* involves inflammatory signaling, mucosal defense and the regulation of innate pattern-recognition pathways. The present transcriptomic results extend these gene-specific observations by revealing coordinated changes in transcripts associated with Toll-like receptor, NF-κB, MAPK and IL-17 signaling in the spleen. Nevertheless, direct comparisons among studies should be made cautiously because of differences in host background, infection route, bacterial dose, tissue and sampling time. Moreover, pathway enrichment reflects coordinated transcriptional changes but does not by itself demonstrate corresponding changes in pathway activity, immune-cell composition or protein function.

Overall, the controlled challenge revealed distinct but partly overlapping transcriptional patterns in the liver and spleen. The liver response was characterized by changes in lipid-related transcripts and apoptosis-associated regulation, whereas the spleen showed broader changes in immune-related pathways. RT-qPCR analysis of eight selected transcripts showed the same expression directions as the RNA-seq data in both tissues, providing additional support for the consistency of the transcriptional results. Together with the clinical and pathological observations from the natural outbreak, these findings provide a clearer picture of *S. iniae*-associated disease in hybrid sturgeon and identify several pathways and transcripts for further investigation. Their specific functional roles, however, remain to be established through experiments at additional time points and at the protein or pathway-activity level.

## 5 Conclusion

This study characterized *S. iniae*-associated disease in farmed hybrid sturgeon (*A. baerii* ♀ × *A. schrenckii* ♂) by combining the investigation of a natural outbreak with a controlled experimental challenge. The isolate recovered from naturally diseased fish was identified as *S. iniae* based on phenotypic characteristics, morphological and ultrastructural observations and *16S rRNA* gene phylogenetic analysis. Histopathological and ultrastructural examinations revealed extensive tissue lesions and cellular injury in the liver, spleen, spiral valve intestine, heart, and gills. In the controlled challenge, PacBio SMRT sequencing provided a full-length transcript-level reference, while Illumina RNA-seq revealed distinct transcriptional responses in the liver and spleen at 48 h post-injection. The liver showed changes mainly involving lipid-related transcripts and apoptosis-associated regulation, whereas the spleen exhibited broader changes in immune-related pathways, including transcripts annotated to the Toll and Imd and IL-17 signaling pathways. RT-qPCR analysis of eight selected transcripts showed expression patterns consistent with the RNA-seq results. Overall, the pathological findings from the natural outbreak and the transcriptional responses observed in the controlled challenge provide complementary evidence for understanding *S. iniae*-associated disease in hybrid sturgeon and identify several pathways and transcripts that warrant further functional investigation.

## Data Availability

The datasets presented in this study can be found in online repositories. The names of the repository/repositories and accession number(s) can be found in the article/**Supplementary Material**.
