# Supplementary material for: A Streptococcus iniae-associated outbreak in hybrid sturgeon (Acipenser baerii ♀ × Acipenser schrenckii ♂): pathological features and transcriptomic responses to experimental challenge
Source: Front Immunol. 2026 Sep 9;17:1893463. doi: 10.3389/fimmu.2026.1893463 (PMC13597461; doi:10.3389/fimmu.2026.1893463)
Supplement: Supplementary file 1 [file DataSheet1.docx]

| **Table S1** Blood agar base composition (per Liter). | |
| --- | --- |
| Ingredients | Amount per Liter |
| Pancreatic digest of casein | 10.0 g |
| Pancreatic digest of heart tissue | 3.0 g |
| Corn starch | 1.0 g |
| Peptic digest of animal tissue | 5.0 g |
| Yeast extract | 5.0 g |
| Sodium chloride | 5.0 g |
| Agar | 15.0 g |
| Defibrinated sheep blood, sterile | 50.0-100.0 mL |
| Distilled water | 1000 mL |
| Final pH | 7.3 ± 0.2 |

| **Table S2** Gene-specific primers for RT-qPCR validation. | | | |
| --- | --- | --- | --- |
| **Gene** | **Primer** | **Sequence (5′ to 3′)** | **Product size (bp)** |
| *FTH1* | F | TGGACATGCACAAGGTGGCATCC | 110 |
|  | R | CCAGTCTCCCAGCTCCTTGATAG |  |
| *GPX1* | F | AAGGACGCAGACCCCATGTTCAC | 111 |
|  | R | GCAGACGGGGCTCCACACAATAA |  |
| *PRDX5* | F | TGGCTGACCTGTTCAAGGGGAAG | 110 |
|  | R | CTCTGCAGCCTGACTCACAAACC |  |
| *DRAM1* | F | TACCCGTTACACCTGGTGAGTGC | 117 |
|  | R | CTCTGCCTTGGCCTTCAGTGTGA |  |
| *MMP13* | F | TCTTCTGGAGGCAGATCCCTCAG | 110 |
|  | R | GCTGTGCTGGCTCTCATAAGCAG |  |
| *CD74* | F | TTGAGGACACAGCCACAGTGAGC | 110 |
|  | R | CAGGTTGTCCAGCAGAGTCTCGT |  |
| *C1QC* | F | ACTACAATCCGTCGACCGGCAAG | 101 |
|  | R | GACGATGCTGACGCACAGGTGAT |  |
| *FABP7* | F | TGTGCAGAAATGGGACGGCAAGG | 102 |
|  | R | GTTCGGACAGCCTGGACATTACC |  |
| *β-actin* | F | CCTCCATCGTCCACCGTAA | 92 |
|  | R | TTCTTTCTGTTTGAGCATTTCTGA |  |
| *EF1α* | F | AAAAGACCCACATCAACATCGT | 186 |
|  | R | CAGCCTTCAGCTTGTCCAAC |  |

| **Table S3** Biochemical identification of the isolated strain. | | |
| --- | --- | --- |
| **Items** | **Isolated strain** | **Reference strains** |
| Growth |  |  |
| 10 ℃ | + | + |
| 45 ℃ | - | - |
| 6.5% NaCl | - | - |
| 8.0% NaCl | - | - |
| *D*-mannitol | + | + |
| Sorbitol | - | - |
| Trehalose | + | + |
| Arabinose | - | - |
| Raffinose | - | - |
| Ribose | - | - |
| Lactose | - | - |
| Oxidase | - | - |
| Catalase | - | - |
| Arginine | + | + |
| Esculin | + | + |
| V-P test | - | - |
| Hemolysis type | β | β |

| **Table S4** Antibiotic sensitivity of the isolated strain. | | | |
| --- | --- | --- | --- |
| **Types** | **Antibiotics** | **Inhibition Diameter** | **Sensitivity** |
| Penicillins | Ampicillin | 33 | S |
| Cephalosporins | Cefoperazone | 34 | S |
|  | Ceftriaxone | 37 | S |
| Tetracyclines | Minocycline | 39 | S |
|  | Tetracycline | 19 | I |
| Aminoglycosides | Streptomycin | 17 | S |
|  | Kanamycin | 21 | S |
|  | Gentamicin | 22 | I |
|  | Tobramycin | 25 | S |
|  | Spectinomycin | 11 | R |
|  | Amikacin | 12 | R |
| Quinolones | Enrofloxacin | 0 | R |
|  | Levofloxacin | 0 | R |
| Nitrofurans | Nitrofurantoin | 15 | I |
| Macrolides | Erythromycin | 17 | I |
|  | Clarithromycin | 21 | S |
|  | Midecamycin | 18 | I |
| Polypeptides | Polymyxin B | 9 | I |
| Lincosamides | Clindamycin | 20 | I |
| Chloramphenicols | Florfenicol | 23 | S |
| Note: S. susceptible; I. intermediate; R. resistance. S, I, and R classifications were provisionally assigned according to the interpretive criteria supplied by the antimicrobial disk manufacturer. Because species-specific clinical breakpoints are unavailable for aquatic *S. iniae* under the present assay conditions, these classifications should be interpreted as descriptive in vitro categories only. | | | |

| **Table S5** Transcriptome sequencing and assembly information in hybrid sturgeon. | | | | | |
| --- | --- | --- | --- | --- | --- |
| **Sample** | **Raw data (bp)** | **Clean data (bp)** | **Q20 rate** | **Q30 rate** | **GC rate** |
| XZ_C_Li1 | 6,084,213,000 | 5,934,491,898 | 98.88 | 95.44 | 48.42 |
| XZ_C_Li2 | 6,949,932,600 | 6,780,379,527 | 98.82 | 95.27 | 48.16 |
| XZ_C_Li3 | 6,246,316,500 | 6,091,052,454 | 98.77 | 95.15 | 47.71 |
| XZ_C_Sp1 | 7,094,123,700 | 6,914,833,429 | 98.72 | 95.01 | 45.49 |
| XZ_C_Sp2 | 6,374,752,500 | 6,206,234,195 | 98.75 | 95.24 | 44.23 |
| XZ_C_Sp3 | 6,703,817,100 | 6,525,847,146 | 98.84 | 95.47 | 45.55 |
| XZ_S_Li1 | 6,734,391,900 | 6,560,245,996 | 98.92 | 95.47 | 45.78 |
| XZ_S_Li2 | 6,306,089,700 | 6,144,405,688 | 98.80 | 95.35 | 45.34 |
| XZ_S_Li3 | 5,690,073,900 | 5,534,270,293 | 98.88 | 95.50 | 46.43 |
| XZ_S_Sp1 | 7,499,658,600 | 7,286,910,390 | 98.88 | 95.62 | 45.19 |
| XZ_S_Sp2 | 6,445,449,900 | 6,273,553,659 | 98.92 | 95.65 | 46.78 |
| XZ_S_Sp3 | 7,110,841,500 | 6,902,784,850 | 98.85 | 95.53 | 46.24 |

**Figure S1** RT-qPCR validation of DEGs in the liver and spleen.
